# The immediate social environment dynamically shapes movement and foraging in wild Sumatran orangutans (*Pongo abelii*)

**DOI:** 10.1186/s40462-026-00713-2

**Published:** 2026-10-21

**Authors:** Emma Lokuciejewski, Margaret Crofoot, Maria van Noordwijk, Odd Jacobson, Fitriah Basalamah, Sri Suci Utami Atmoko, Caroline Schuppli

**Affiliations:** 1 Development and Evolution of Cognition, Max Planck Institute of Animal Behavior, Konstanz, Germany; 2 Department of Biology, University of Konstanz, Konstanz, Germany; 3 International Max Planck Research School for Quantitative Behaviour, Ecology and Evolution, Konstanz, Germany; 4 Department of the Ecology of Animal Societies, Max Planck Institute of Animal Behavior, Konstanz, Germany; 5 Comparative Socioecology Group, Max Planck Institute of Animal Behavior, Konstanz, Germany; 6 Faculty of Biology and Agriculture, Universitas Nasional, Jakarta, Indonesia

**Keywords:** Costs and benefits of associations, Movement patterns, Foraging behaviour, Spatial ecology, Daily path length, Sinuosity, Fission-fusion dynamics

## Abstract

**Background:**

Social associations have well-established costs and benefits, and likely play a fundamental role in shaping animals’ movement. Individuals are predicted to balance the costs of associating, such as feeding competition, against key benefits, including social learning and mating opportunities, resulting in context-dependent movement patterns. However, the effects of the immediate social environment on movement remain poorly understood, likely because most movement ecology research focusses on either strictly group-living or solitary species. We investigate how social associations shape fine-scale movement patterns and food resource exploitation in wild, semi-solitary Sumatran orangutans (*Pongo abelii*) who show fission-fusion social dynamics. We integrated 16 years of spatial data on 72 individuals with detailed behavioural observations. We predicted that associations shape movement in ways that reflect distinct costs and benefits across age-sex classes.

**Methods:**

We quantified the effects of associations on daily path length (a proxy for energetic investment in movement) using a continuous-time speed and distance (CTSD) approach, path sinuosity (as a measure of movement efficiency), and the use of key food hotspots (as an indicator of feeding competition and spatial knowledge). Using linear mixed models, we tested how these metrics varied with time spent in association and age-sex class, controlling for ecological factors.

**Results:**

Across all age-sex classes, daily path length increased with time in association, driven by longer active periods. At the population level, there was a trend towards straighter paths when individuals were with associates, though this depended on the class of the focal and the associate. Foraging behaviour was socially sensitive, with all age-sex classes visiting fewer feeding hotspots when in associations, and spending less time feeding within them. Social influences on movement and foraging were most apparent in mothers, although flanged males also exhibited significant behavioural effects, including the greatest reduction in overall feeding time when in the presence of unflanged males compared to when alone.

**Conclusions:**

Together, these results suggest that social associations entail costs and benefits for orangutans that are tangibly reflected in their movement and foraging decisions. Furthermore, different age-sex classes showed distinct adjustments in movement depending on who they associated with, highlighting the dynamic nature of social influences on behaviour.

**Supplementary Information:**

The online version contains supplementary material available at https://doi.org/10.1186/s40462-026-00713-2.

## Background

Animal movement is shaped by an interplay of social and ecological factors, as well as individual- and group-level characteristics [1, 2]. Movement ecology seeks to understand how these interacting factors generate patterns of movement across space and time that reflect evolved adaptive responses [3]. Among these factors, sociality plays a central role: at the species level, it can facilitate or constrain individuals’ spatial decisions, while within species the immediate social environment (e.g., the occurence and duration of associations with individuals of different age-sex classes) may both drive movement and emerge as a consequence of individuals’ movement trajectories as they balance experienced costs and benefits [4]. The reciprocal relationship between the immediate social environment and movement remains poorly understood, largely owing to the complexities of social contexts that may influence individuals differently. Moreover, most movement ecology research has focused on species that are either strictly group-living or strictly solitary.

Orangutans represent a particularly informative system for disentangling the interplay between the immediate social environment and movement because of their flexible and context-dependent sociality. Orangutans are unique among great apes in exhibiting characteristically low and dynamic tendencies to associate with conspecifics, typically described as a semi-solitary fission-fusion dynamic [5–7]. This social system has been attributed to a combination of high energetic need and a predominantly frugivorous diet [8], which together impose a high caloric need in a habitat characterised by temporally fluctuating and spatially patchy food resources [9]. Despite being less gregarious than African great apes [10, 11], orangutans do nonetheless associate more frequently than expected by chance [7, 10, 12, 13], suggesting that social factors play a meaningful role in shaping their spatial ecology.

Orangutan associations may be passive in nature, involving no direct social interactions, commonly observed at foraging sites [12], or active, involving coordinated movement and social attraction [12, 14]. They vary in size (i.e., the number of individuals involved) and composition (i.e., the identities of those individuals) and range from brief encounters lasting only minutes, to stable associations persisting over multiple consecutive days [5, 13, 15]. During longer encounters, individuals have more opportunities to travel together, share feeding sites, and engage in social interactions [12]. In orangutans, such associations have been linked to increased mating opportunities [5], and enhanced opportunities for social learning [16]. Association frequencies and sizes systematically vary across populations [7, 10], age-sex classes [5, 17], reproductive state [18], and habitat productivity [10, 19], likely reflecting a dynamic balance between ecological and social costs and benefits.

Orangutans are subject to pronounced energetic constraints arising from their relatively large brains, large body size, energetically costly arboreal locomotion [20], and residence in low-food-density habitats that limit energy acquisition. Together, these factors place individuals under persistent energetic pressure, requiring careful trade-offs in how energy is allocated, particularly with respect to movement. Given that locomotion in orangutans is already energetically demanding, deviations from energetically efficient movement due to the presence of conspecifics likely generate costs [15, 21] that must be offset by social benefits [22]. Within this context, associations can provide important social benefits, such as facilitating mating [23], enabling social learning [16, 24], and providing play opportunities for immatures [25]. However, associations can also impose costs, such as increased feeding competition [26], forced copulations [18], and potential infanticide risks [18]. Previous studies further suggest that having more associates present increases an orangutan’s travel distance [5, 15, 27], though these studies acknowledge that individuals may differ in their intrinsic costs, such as direct locomotor costs and nutritional needs [5]. Conversely, avoiding associations with conspecifics may alleviate costs, but also reduce beneficial opportunities. Taken together, the infrequency and conditional nature of orangutan associations and social interactions seem to reflect a trade-off between ecological constraints, energy expenditure, and the potential advantages of sociality.

The aforementioned costs and benefits of associations are predicted to shape movement decisions by influencing when orangutans tolerate or seek proximity to others versus when they prioritize being on their own and avoid associations. Each age-sex class of orangutan likely experiences a different balance of these costs and benefits.

Oestrus females without dependent offspring actively seek mates, with associations primarily occurring for mating opportunities, however this can expose them to increased feeding competition, and also harassment from coercive mating attempts [18, 23, 28]. Mothers, in contrast, maintain prolonged associations to their dependent offspring [29, 30], and seemingly adjust movement to reduce risk of harassment to the infant [5, 31, 32], prioritising offspring safety [18, 31] over social opportunities for herself [31, 33] and for her infant [34]. Flanged males, with their larger body size and secondary sexual characteristics, can monopolise access to food resources and females, influencing the movement of others [15, 35], such as attracting females [36], and displacing other males [35]. However, because of their large body size, they face more pronounced energetic costs [36], which may limit their travel [9]. Unflanged males, on the other hand, being smaller, have lower energy demands, allowing them to allocate more energy towards searching for females.

Adult females and flanged males are often resident individuals, and therefore likely benefit from their greater knowledge of the spatial distribution of resources, and social dynamics. Unflanged males are more likely to be new to an area because of recent dispersal from their natal range leading to reduced spatial knowledge. Unflanged males are thus predicted to benefit from associating with more familiar individuals to acquire information on resource locations and social dynamics [37, 38], although this entails risks such as displacement by dominant males and increased competition. Immature individuals, who are therefore likely to be more naïve are expected to benefit from associations by gaining social learning opportunities [39, 40], important for the development of ecological competence [24, 41]. However, they may face increased energetic costs associated with increased exploratory behaviour, and likelihood of displacement by residents [37].

Across all classes, these trade-offs are likely to be context-dependent, with greater social tolerance at high-value (high abundance) feeding sites, whereby feeding competition and energetic costs are balanced by nutritional gains and temporary social advantages [19, 42].

Studying spatial and foraging metrics offers a powerful tool for examining the costs and benefits of social associations. Previous research on primate movement ecology has demonstrated the influence of social factors on travel speed [43–45], route choice [46], foraging patch visitation [47], and resource competition [48]. However, these studies have typically focused on relatively broad social influences, such as group size, group cohesion and collective movement of group-living species. Research on orangutan movement in particular has primarily focussed on individual-level spatial metrics, such as home ranges [49–54], and daily path lengths [49, 51], treating individuals as solitary units, rather than temporally associating conspecifics and neighbours. As a result, few empirical studies have quantified how associate age-sex class shapes spatial metrics in semi-solitary species such as orangutans, meaning the social drivers of these unique and ephemeral interactions are therefore often overlooked.

In this study, we address this knowledge gap by investigating how associations among different age-sex classes, and the time spent together (termed the immediate social environment), shape movement patterns and visitation of high-value food sites of wild Sumatran orangutans (*Pongo abelii*). Specifically, we quantify spatial metrics - measurable properties of how individuals move through and use space, including the length and shape of daily travel paths and the use of high-value foraging locations. We then examine how the presence of mothers, independent immatures, flanged males, and unflanged males influences these spatial metrics. By integrating fine-scale movement data with simultaneous behavioural observations, we aim to identify how the immediate social environment modulates movement behaviour. In doing so, we provide new insight into the costs and benefits of sociability.

Based on the age-sex-class-specific costs and benefits of associations outlined above, we hypothesize that associations will differentially shape orangutan movement and foraging behaviour depending on the specific immediate social environment. As a result, we predict that associations will alter the energetic investment in movement, travel efficiency, and patterns of resource use. We expect the nature of these responses to balance the effects of shared spatial knowledge, mating strategies, social dominance, and feeding competition.

In particular, we predict that longer associations will generally increase movement costs, such as more time spent moving and greater distance travelled, and reduce travel efficiency overall due to increased opportunities for adjusting movement in response to associates, including both following and avoiding them, whereas solitary conditions should favour more direct and energy-efficient movement patterns. However, the direction and magnitude of these effects will vary depending on the age-sex class combination of focal and associate, and will shift as a function of time spent in association.

We predict that individuals with less spatial knowledge of the area, such as unflanged males and immatures, will benefit from associating with more knowledgeable associates, such as flanged males and mothers. These associations are therefore expected to improve resource acquisition by facilitating access to profitable feeding areas while reducing unnecessary search effort. We also predict that knowledgeable individuals will more frequently lead movement bouts when travelling with an associate.

We further predict that mothers, because of their need to avoid coercive mating attempts by unflanged males, would show more direct travel to increase distance from unwanted associates. Consequently, we expect mothers to more frequently lead movement bouts during these associations, with unflanged males following females in an attempt to maintain proximity. Associations with flanged males are expected to have individual specific effects, such as seeking established, locally dominant flanged males, but avoiding newly immigrated or non-resident flanged males.

Associations between males are expected to have weaker or contrasting effects due to dominance-related displacement; when encountering more dominant rivals, displacement may cause greater movement costs and reduced opportunities to exploit food resources, whereas more dominant males should retain access to preferred feeding areas during associations.

Finally, we predict that increased time spent with associates will increase scramble competition, leading to accelerated local resource depletion and thus shorter time spent in feeding patches, as well as an increased need to seek alternative resources. These effects are expected to be strongest among associations involving competitors (e.g., flanged males associated with other males) or vulnerable individuals (e.g., mothers associated with unflanged males and non-resident flanged males), whereas associations with more tolerant or knowledgeable individuals are expected to show weaker effects because they allow for more shared space use (e.g., immatures associated with mothers or flanged males).

## Methods

### Data collection

Between 2007 and 2023, we conducted focal follows on wild Sumatran orangutans (*Pongo abelii*) at the Suaq Balimbing field site within the Gunung Leuser National Park, in South Aceh, Indonesia (97.431°E, 3.050°N). Data collection was conducted by 62 observers over the study period, with new observers training with experienced observers until an inter-observer reliability score of 80% was met, to ensure consistency in behavioural observations. We located focal individuals opportunistically by systematically traversing predefined transects within the study area, while actively surveying for recent indicators of orangutan presence (e.g., feeding traces, nests), and auditory cues such as vocalizations or canopy movement. We recorded behavioural data at 2-minute intervals during focal follows using standardised behavioural ethograms [55], and we logged corresponding GPS locations at 10-minute intervals, using handheld GPS devices directly below the focal. Observers always remained a minimum of 10 m away from the focal, and GPS waypoints were taken at the nearest feasible opportunity with timepoints adjusted retroactively if the focal individual climbed down lower in the trees where maintaining the required distance could no longer be ensured. Whenever possible, follows covered the full daily active period of orangutans, from departure from the sleeping nest in the morning to the construction of a new nest in the evening (nest-to-nest follows). The average duration of nest-to-nest follows, representing the individuals’ active period, was 11.28 h (SD = 1.00, range = 4.90 to 13.57). Most individuals were fully habituated to human observers, and we observed no apparent behavioural responses to observer presence for any of the included focal animals, including among newly observed individuals, such as typically less-habituated unflanged males.

Our data totalled 1235 nest-to-nest behavioural observations (follows) collected from 110 individuals. We categorised individuals into four age-sex classes following established classifications widely used in orangutan research, based on morphological characteristics, developmental stage, and reproductive status [9, 36]; unflanged males (adult males exhibiting arrested development), flanged males (adult males with secondary sexual characteristics such as cheek flanges), mothers (adult females with dependent immatures), and independent immatures (who range independently from their mother). Immature individuals were classified as independent when they spent three consecutive days away from their mother, signalling social and nutritional independence. We excluded dependent immatures, unidentified individuals, and adult females of reproductive age without offspring, due to the brief duration of this reproductive state and insufficient sample size. Our dataset included behavioural data on 39 unflanged males, 27 flanged males, 30 independent immatures, and 22 mothers. Of these, 72 individuals also had corresponding GPS data, comprising 20 unflanged males, 18 flanged males, 22 independent immatures, and 16 mothers. Over the course of the study, eight individuals were observed to transition between age-sex classes, including 3 individuals transitioning from unflanged male to flanged male, and 5 from independent immature to mother.

We considered associates present when they were located within 50 m of the focal individual, a range that generally permits visual contact despite dense foliage [55]. To characterise behaviour during associations, we also investigated behaviours occurring during the recorded associations. We calculated activity budgets by summarising the percentage of time the focal spent feeding, resting, moving, nesting, socialising, and engaging in other behaviours. For each follow, we calculated the proportion of time allocated to each activity category within each focal–associate age-sex class combination, and also when alone. These proportions were then averaged across follows within each focal–associate class combination, such that each follow contributed equally to the resulting activity-budget estimates.

We also quantified movement order, by identifying which individual initiated and which followed in each movement bout to distinguish individuals seeking social opportunities from those serving as the target. A movement bout was defined as a continuous period during which the focal individual was engaged in travel between locations, ending when movement ceased and the individual transitioned to another behavioural state (e.g., feeding, resting, socialising, or nesting). For each follow, we calculated the daily proportion of bouts in which the focal led or followed, then averaged these values across age-sex class dyads to ensure equal weighting. By linking movement and foraging behaviour to the social context in which it occurred, we provide a rare test of how the presence and class of associates modulate animal decision-making, providing additional context for interpreting the results.

We measured daily rainfall in millimetres, using a graduated measuring cylinder at a consistent open location at the research station, recorded at the same time each morning and evening. Our analyses used the evening record representing rainfall accumulated during the daytime, therefore the orangutan’s active period. We quantified fruit availability through monthly phenology surveys identifying fruit-bearing trees, performed along predefined transects spanning north to south and west to east throughout the study site, encompassing approximately 1000 trees with a diameter at breast height of > 20 cm (Supplementary Fig. 1). We calculated the proportion of fruiting trees and averaged this value to provide a monthly site-wide index of fruit availability [9].

### Response metrics

We quantified movement behaviour using four complementary metrics, each capturing a different aspect of spatial ecology: (1) daily path length (DPL) as a proxy for overall energetic investment in daily movement effort, (2) path sinuosity as a measure of movement efficiency, indicating whether travel was goal-directed or exploratory, (3) the number of feeding hotspots visited as a measure of key resource identification and utilisation, providing insights into spatial knowledge and potential cognitive maps, and (4) time spent in the feeding hotspot to assess resource exploitation and displacement from the hotspot. Together, these metrics link energy expenditure with spatial decision-making and resource use strategies.

### Daily path length (DPL)

We estimated daily path lengths using the continuous-time speed and distance (CTSD) approach [56] implemented within the continuous-time movement modelling (CTMM) framework of the *ctmm* R package [57]. Unlike conventional approaches that sum straight-line distances between successive GPS fixes, CTSD fits a continuous-time movement model to estimate mean speed, which is scaled by the daily observation duration to obtain distance travelled. This approach is robust to variation in sampling rate and path tortuosity [56]. To reduce inflation from GPS error, we incorporated an error model with a weakly informative prior on the root mean square (RMS) user equivalent range error (UERE) centred at 15 m (95% credible interval: ~2–29 m), allowing error variance to be estimated jointly with movement parameters [58]. For each follow-day, the best-supported model was selected using AIC (*ctmm.select*), and daily path length was calculated as mean speed multiplied by observation duration.

### Sinuosity

Sinuosity is a measure of path tortuosity derived from the directional persistence and step length variation of the trajectory [59], where higher values indicate increasingly tortuous movement. Values were calculated using the *trj_sinuosity2* function in the ‘trajR’ package [60].

$$\:Sinuosity=2\:[p\left(\frac{1+c}{1-c}\right)+{b}^{2}{]}^{-0.5}$$

Where *p* is the mean step length, *c* is the mean cosine of turning angles, and *b* is the coefficient of variation of the step length [59]. This method provides a corrected sinuosity index that generalizes the original formulation by Bovet & Benhamou (1988) [61] to accommodate a wider range of turning angle distributions and variable step lengths.

### Number of feeding hotspots visited per day

The number of feeding hotspots visited per day was calculated as the total number of distinct feeding hotspots visited by each focal individual during each nest-to-nest observation. We defined feeding hotspots as individual trees or small clusters of trees (within a 15 m crown radius) where the focal individual remained feeding continuously for more than 30 min.

### Time (minutes) spent feeding in a hotspot

Time spent feeding in a hotspot was calculated as the total duration (minutes) that focal individuals spent feeding within a defined hotspot. Continuous feeding bouts were operationally defined as consecutive sampling points in which the focal individual was observed feeding, with patch entry and exit also recorded to validate distinct feeding bouts. The distribution of feeding bout durations was strongly right-skewed, with a mean of 22 min and a modal duration of 9 min, revealing two distinct behavioural patterns; brief sampling, often whilst travelling, and prolonged feeding. Based on this distribution, we applied a conservative threshold of 30 min to define a feeding hotspot, as extended feeding time likely reflects access to a particularly abundant and/or favourable food source, when time spent feeding is used as a proxy for item abundance and/or value. 20.1% of feeding bouts lasted longer than 30 min.

### Analyses

We analysed DPL, sinuosity and number of feeding hotspots per day using full-day nest-to-nest focal follows. For analyses of time spent in each hotspot, we also included shorter observation days to increase sample size, as these still captured complete feeding bouts within hotspots. For analyses of path length and sinuosity, we included only focal follows with ≥ 75% GPS waypoint completeness, to ensure paths were sufficiently complete and not biased by large temporal gaps. To evaluate the robustness of this threshold, we conducted a sensitivity analysis in which the daily path length and sinuosity models were repeated using alternative GPS completeness thresholds (≥ 25%, ≥ 50%, ≥ 75%, and 100%). We compared changes in sample size and examined whether the estimated effects of time spent in association on daily path length and path sinuosity remained consistent across thresholds (Supplementary Table 1). Lower completeness thresholds allow higher data retention but increase the likelihood that movement paths are affected by missing locations and uneven sampling intervals, whereas a 100% completeness requirement substantially reduces the number of available follows and focal individuals, potentially limiting statistical power. The ≥ 75% threshold therefore represented a compromise between trajectory reliability and sample retention. The mean number of waypoints in a daily path with > 75% GPS completeness was 66 (SD = 7, range: 39 to 83).

For each observation day with sufficient GPS data, we calculated the total number of GPS waypoints recorded at fixed 10-minute intervals as an index of sampling density, and included this variable as a control in analyses of sinuosity to account for variation in track resolution. Because sinuosity metrics depend on sampling density, we expected a greater number of waypoints to inflate estimated sinuosity, as additional points capture more small-scale turns and deviations along the path. In analyses of hotspot use, we controlled for active period duration (hours), as longer follows provide greater opportunity for individuals to encounter and exploit feeding hotspots. Path length estimates from CTSDs already account for sampling density.

We systematically recorded the presence or absence of associates from each of the four age-sex classes, at each 2-minute sampling interval. This allowed us to calculate, for each age-sex class separately, the proportion of time that a focal individual was associated with that class, across the full nest-to-nest follow, resulting in one data point per focal per day. Because feeding hotspots represent subsets of the total daily observation period and multiple hotspots could be visited within a single follow, we additionally calculated associate presence/absence and proportion of time in association separately for each hotspot visit. This distinction was necessary because associates were often only present during part of the day; therefore, an individual could be associated with a focal during one hotspot visit but absent during another, resulting in differences between overall daily association measures and hotspot-specific association patterns. We use the term immediate social environment to refer to the presence and duration of associations with individuals from each of the four age-sex classes, measured independently. Importantly, the immediate social environment was not treated as a single composite group-level variable, nor did we explicitly quantify overall social group size; rather, it was operationalized through independent measures of association with each age-sex class. Multiple associate classes could be present at the same time and were therefore recorded independently. For example, if an unflanged male and a mother were both present within 50 m of a focal individual for 30 min, the focal was recorded as associated with an unflanged male for those 30 min and as associated with a mother for those same 30 min. Thus, the same period could contribute to the association measure for more than one age-sex class.

Because multiple associate classes could be present simultaneously, we assessed multicollinearity among predictors in the analyses testing the effects of specific associate classes on movement and foraging. Model diagnostics across all predictors indicated low variance inflation factors (VIFs), ruling out problematic collinearity among all predictors and confirming that the effects of different associate classes could be estimated independently despite their potential co-occurrence.

**Fig. 1:**
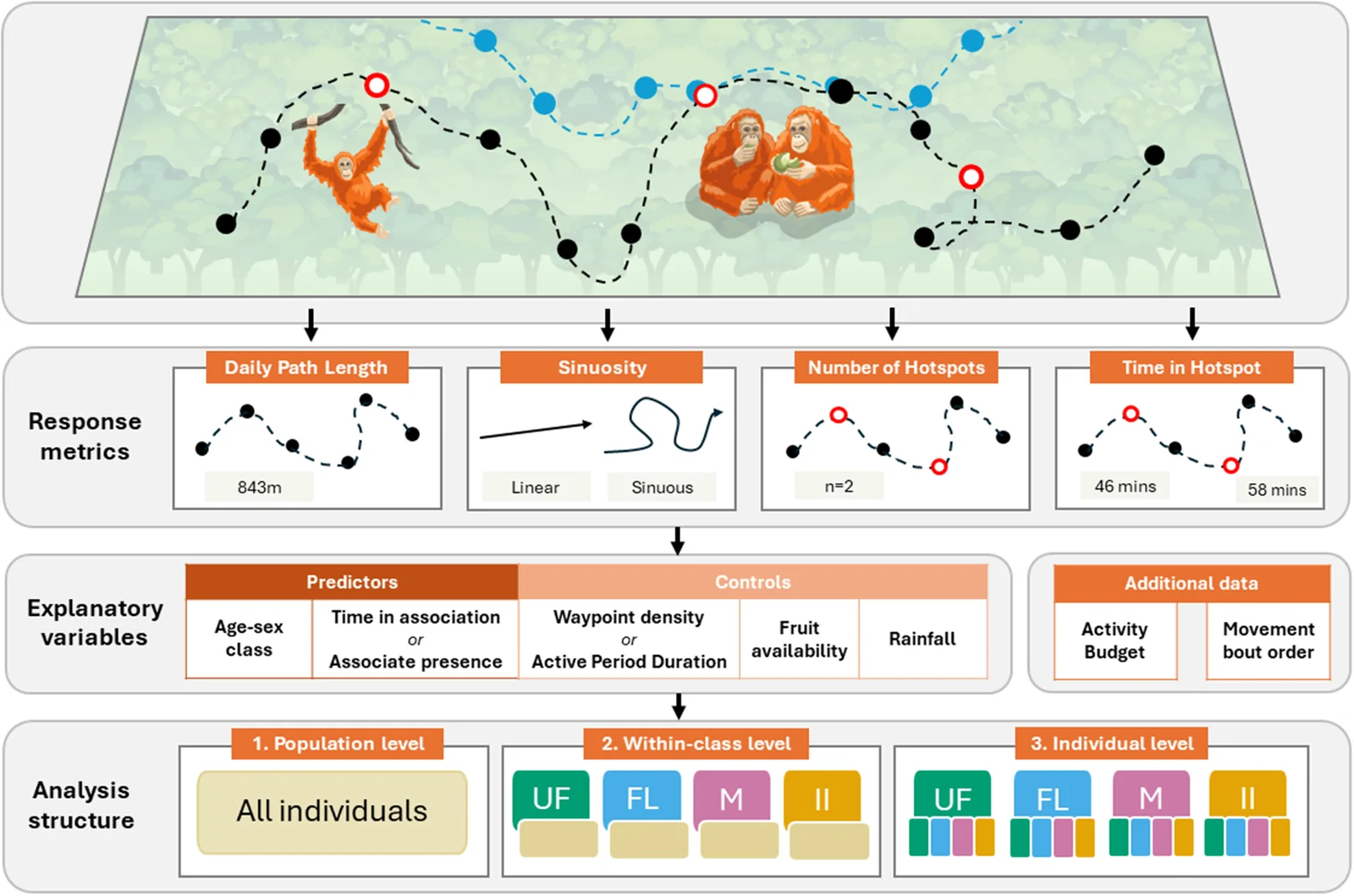
Conceptual overview of an orangutan’s daily movement and the analytical framework we used to analyse the effects of associations. Response metrics include daily path length (DPL), sinuosity, number of feeding hotspots visited, and time spent within a hotspot. The three-tiered analysis structure is shown whereby UF=Unflanged males, F=Flanged males, M=Mothers, and II=Independent immatures

We implemented a three-tiered analytical framework to examine movement and foraging patterns at three different scales considering focal age-sex class, immediate social environment, and ecological factors (Fig. 1). Using linear mixed models, we tested how associations influenced the four behavioural metrics. We first analysed the effects of associations on behavioural metrics across all focal age-sex classes, irrespective of associate class, which also allowed us to compare differences in spatial metrics across all focal age-sex classes and the effects of other contextual variables (Population-level). Secondly, we ran separate models for each behavioural metric and each focal age-sex class to capture within-class variation in effects of associations (Within-class level). This approach acknowledges that behavioural responses may not be uniform across age-sex classes, and it provides class-specific estimates while retaining the same set of predictors used in the population-level analyses. Third, we evaluated how the presence of associates from each age-sex class influenced the movement and foraging of each class of focal (Focal-associate class combination level). For population and within-class levels we included proportion of the observed period spent in association as a predictor. For the focal-associate class combination level analyses we used presence and absence of an associate (at the daily level, and at the hotspot-specific level), rather than duration of the association as this approach was more robust given limited sample sizes of some rare dyad classes (Supplementary Table 2). These models tested the independent effects of each associate age-sex class. This approach is based on the assumption that mere presence of the associate can alter movement, regardless of association duration. Population-level analyses involved the largest sample sizes and thus provided more robust population-level estimates. Building on this, within-class analyses allowed social and ecological predictors to have class-specific effects and captured heterogeneity in behavioural responses. The focal-associate class combination level analyses added critical understanding of dyad-specific variation, helping to explain the patterns detected at the prior levels.

The four aforementioned behavioural metrics were the response variables in the models. Full models were fitted including the factors of age-sex class, percentage of time in association, or associate presence, as predictors, in addition to control variables: fruit availability, rainfall, and, depending on the response variable, number of GPS waypoints, or active period duration. We used mixed-effects models to account for non-independence of repeated measures. Random intercepts for focal ID accounted for repeated measures of individuals, and follow ID for multiple measures of hotspots per day. We used linear mixed models (LMMs; lmer) for the response variables DPL, sinuosity, and log-transformed time spent in a hotspot as they were continuous and approximately normally distributed. For the number of hotspots visited per day (as a count variable), we used generalised linear mixed models (GLMMs; glmmTMB) with a Conway-Maxwell Poisson distribution. We used packages ‘lme4’ [62] to fit LMMs, and ‘glmmTMB’ [63] to fit GLMMs, with appropriate diagnostic checks performed to verify model assumptions (residual patterns, homoscedasticity, model specification, and potential influential observations using check_model(), and multicollinearity among predictors using check_collinearity(), from the ‘performance’ package [64]). Detailed model structures and sample sizes are provided in Supplementary Tables 2, and model outputs provided in Supplementary Table 3.

All analyses were conducted in R [65]. We used Likelihood Ratio Tests (LRTs) via the *anova()* function to compare full models (containing all predictors and random intercepts) to null models (containing only random intercepts). We assessed significance of fixed effects using Type III Wald χ² tests, and we conducted Tukey-adjusted post-hoc pairwise comparisons [66] to evaluate differences among age-sex classes.

## Results

**Fig. 2:**
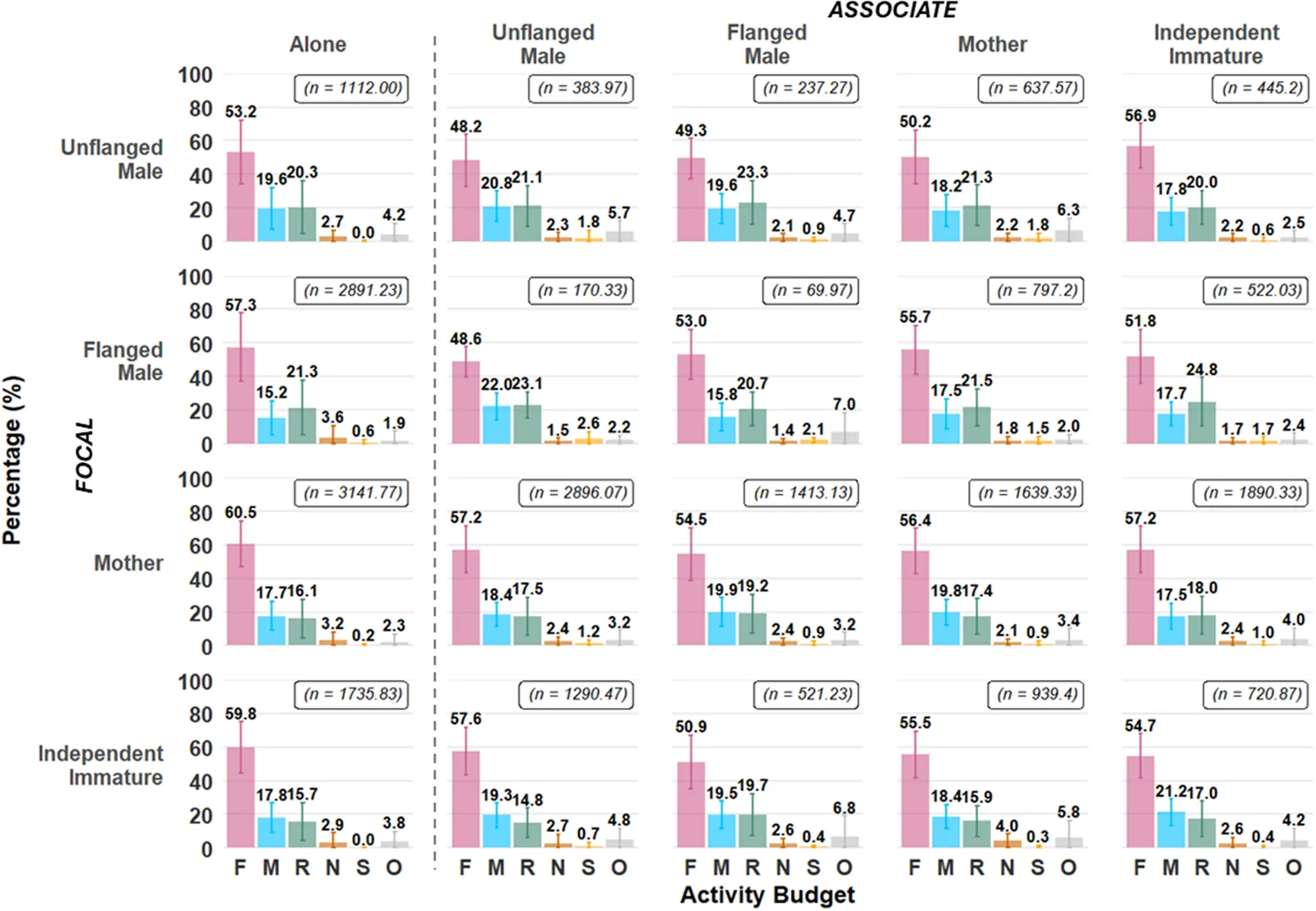
Mean activity budgets during associations with different associate classes compared to solitary periods, across focal-associate dyad types. Each cell in the matrix represents either a focal individual when alone, or a focal-associate class combination, and shows the activity budget of the focal individual. Bars indicate the mean percentage of time spent in each activity: feeding (F), moving (M), resting (R), nesting (N), social (S), and (O) other, per active period of a full-day observation. Social behaviours observed when alone refer to the long call vocalisations made by flanged males, and social behaviour from mothers to their dependent offspring (who are not considered an associate). Error bars represent SDs calculated across follows. n = total hours of observation per association category

Activity Budget: Activity budgets revealed differences in how focal individuals allocate time across behaviours depending on the age-sex class of their associates, or whether they were alone. Feeding was the most prevalent behaviour across all focal individuals and dyads; although proportions declined during association (Fig. 2*)*. Flanged males exhibited the largest behavioural shifts between solitary and social conditions, whereas the magnitude was smallest for mothers. In particular, flanged males displayed the greatest reduction in feeding when associated with unflanged males (57.3% when alone to 48.6% when associated), accompanied by an increase in movement (15.2% when alone to 22.0% when associated). Independent immatures showed increased movement with every associate age-sex class. Social interactions were generally rare, with the lowest levels in independent immatures (0.3–0.7%), and highest when flanged males associated with unflanged males (2.6%), primarily in the form of social watching and antagonistic behaviours towards the associate.

**Fig. 3:**
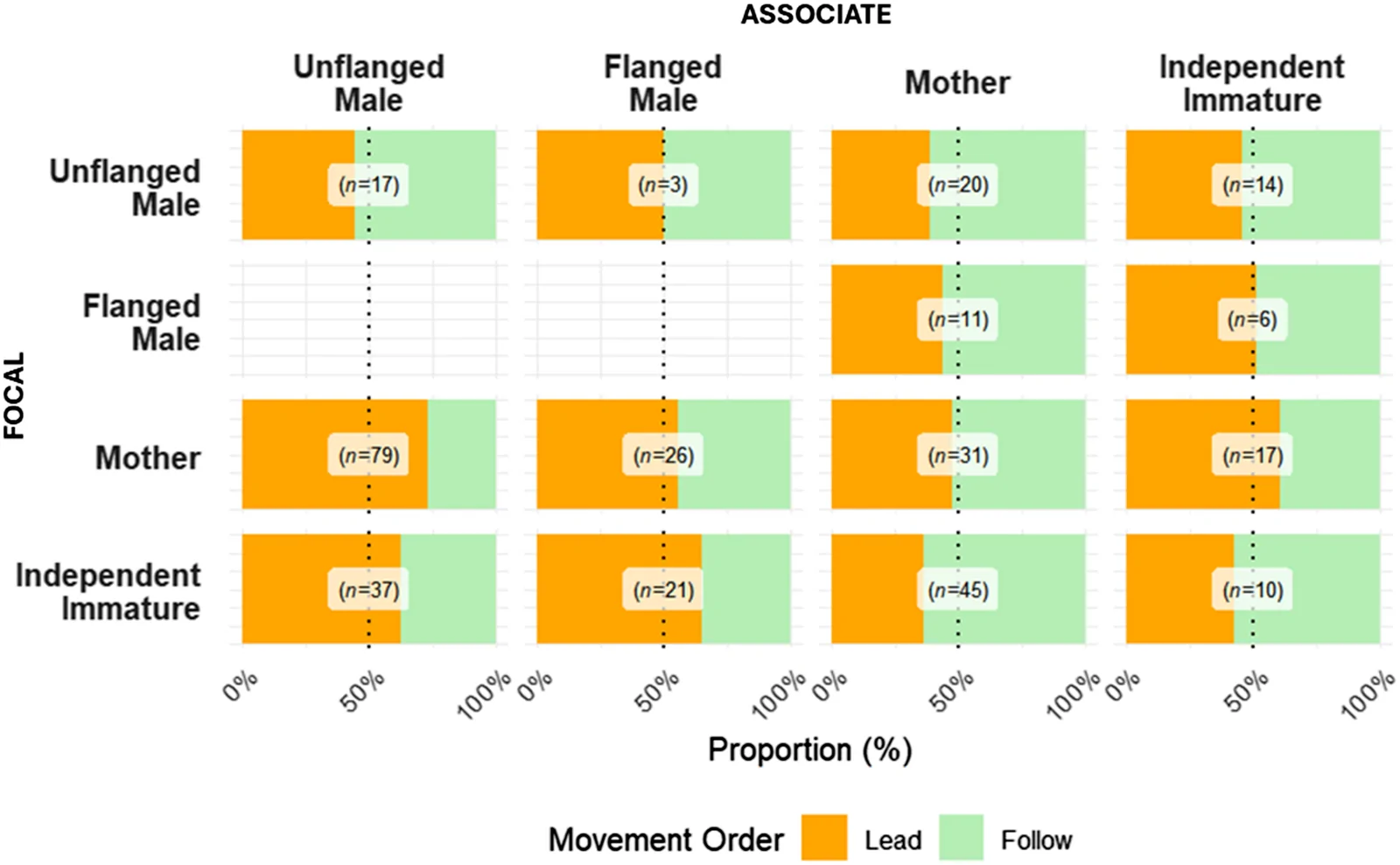
Movement order across focal-associate dyad types. Each cell in the 4 × 4 matrix represents a focal–associate class combination and shows lead-follow movement dynamics. Stacked bars indicate the proportion of time the focal individual leads or follows movement bouts with the associate, per active period of a full-day observation. The dotted line at 50% indicates equal lead-follow proportions. n = number of observation days per dyad

Movement order: Movement sequence analyses revealed differences in the extent to which focal individuals led or followed movement bouts depending on associate age-sex class. Independent immatures generally led movement when paired with flanged and unflanged males, but tended to follow mothers (Fig. 3). Mothers led movement more often than followed regardless of associate class. Flanged males tended to follow mothers, while unflanged males tended to follow associates from all classes, except in the presence of flanged males, where proportions were more equally balanced.

### Population-level effects of associations and age-sex class on movement and foraging patterns

On average, orangutans at Suaq Balimbing were alone 53.6% of the time, with 1 other individual (excluding dependent immatures) 26.9% of the time, and with 2 or more individuals 19.6% of the time. Independent immatures were the most social class, being with at least one associate 56.1% of the time (including with their own mother 85% of this association time), followed by unflanged males (50.0%) and mothers (46.1%). Flanged males were with an associate only 32.7% of the time. Further population-level and age-sex level summary statistics are found in Supplementary Table 4.

**Fig. 4:**
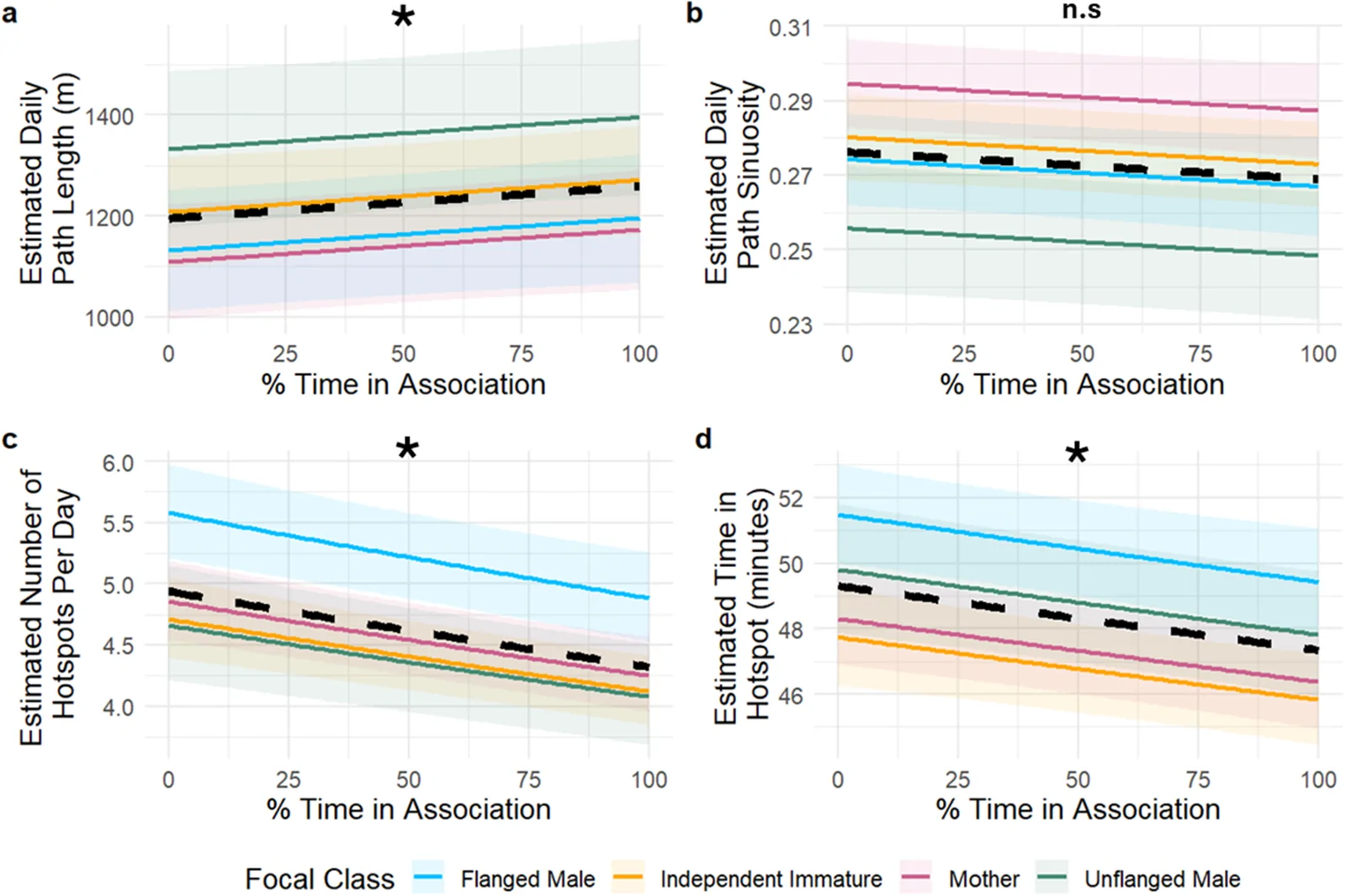
Effects of associations on different spatial metrics of Sumatran orangutans at the population level. Panels show the relationship between the percentage of time spent in association per day and (**a**) daily path length, (**b**) daily path sinuosity, (**c**) number of feeding hotspots visited per day, and (**d**) time spent feeding in a hotspot. A value of 0% time in association represents days when the focal individual was alone. The black dashed line represents the population-level average, while coloured lines denote individual age-sex classes. Lines are derived from model-estimated values with all predictors, except time in association, held at their mean (estimated using the emmeans() function in R), and shaded ribbons indicate 95% confidence intervals. Asterisks represent a significant effect of % Time in association (*p* < 0.05) on the corresponding response metrics, n.s = non-significant model result

Daily path length: Orangutans at Suaq Balimbing travelled 1130 m per day on average (SD = 503) when alone, increasing to 1201 m (SD = 425) when in association for the whole active period. The full model explained significantly more variation in daily path length (DPL) than the corresponding null model (χ²(6) = 18.10, *p* = 0.006). Time spent in association showed a significant positive effect on DPL (χ²(1) = 4.58, *p* = 0.032; Fig. 4.*a*). Age-sex classes had a significant overall effect on DPL (χ²(3) = 8.53, *p* = 0.036); however, none of the pairwise contrasts were significant. Fruit availability had a significant positive effect on DPL (χ²(1) = 3.88, *p* = 0.048). Rainfall did not significantly affect DPL. Complete model outputs are provided in Supplementary Table 3.

To determine whether longer paths were mediated by associate presence leading to longer active periods we re-ran the full model on DPL, with the inclusion of active period as a control variable. In this model, the association between time spent in association and DPL was no longer significant, signifying that individuals that spend more time in association do not travel farther per unit time, but they are active for longer, resulting in greater total movement distances (Supplementary Table 5).

Sinuosity of the daily path: There was a population-level trend of straighter paths when spending more time in association (χ²(1) = 3.76, *p* = 0.053; Fig. 4.*b*). The full model explained significantly more variation in path sinuosity than the corresponding null model (χ²(7) = 63.54, *p* < 0.001). The overall effect of age-sex class on path sinuosity was significant (χ²(3) = 18.44, *p* < 0.001), with mothers (who are by definition always accompanied by a dependent offspring) showing greater sinuosity than unflanged males (mean difference = 0.039, *p* < 0.001); other pairwise contrasts were not significant. Daily path sinuosity increased with the number of waypoints (χ²(1) = 43.80, *p* < 0.001; Supplementary Table 6). Fruit availability and rainfall did not significantly affect daily path sinuosity.

Number of feeding hotspots visited per day: Orangutans visited 5.09 (SD = 1.84) feeding hotspots per day on average when alone, decreasing to 4.45 (SD = 1.85) when in association for the whole active period. The full model provided a significantly better fit for the number of hotspots visited per day compared to the corresponding null model (χ²(7) = 59.957, *p* < 0.001). Time spent in association had a significant negative effect on the number of feeding hotspots visited per day (χ²(1) = 25.79, *p* < 0.001; Fig. 4.*c*). Age-sex class had a significant overall effect (χ²(3) = 15.77, *p* = 0.001), with flanged males visiting more hotspots than independent immatures (rate ratio = 1.18, *p* = 0.003), mothers (rate ratio = 1.15, *p* = 0.021), and unflanged males (rate ratio = 1.20, *p* = 0.014). The number of hotspots visited per day increased significantly with active period duration (χ²(1) = 21.74, *p* < 0.001). Fruit availability and rainfall had no significant effects.

Time spent feeding in a hotspot: Orangutans spent an average of 55 min (SD = 30) feeding in a hotspot when alone, decreasing to 51 min (SD = 27) when in association for the entire hotspot visit, and the magnitude of this varied across age-sex classes. The full model explained significantly more variation in time spent feeding in a hotspot than the corresponding null model (χ²(7) = 34.49, *p* < 0.001). Time spent in association had a significant negative effect on the time spent in a hotspot (χ²(1) = 13.31, *p* < 0.001; Fig. 4.*d*). Age-sex class had a significant overall effect (χ²(3) = 15.12, *p* = 0.002), with flanged males spending significantly longer in a hotspot than independent immatures (ratio = 1.08, *p* = 0.002) and mothers (ratio = 1.07, *p* = 0.009), corresponding to approximately 8% and 7% longer hotspot residence times, respectively. All other pairwise contrasts were not significant. Active period duration, fruit availability, and rainfall did not significantly affect time spent feeding in a hotspot.

### Within-class effects of associations on movement and foraging patterns

**Fig. 5:**
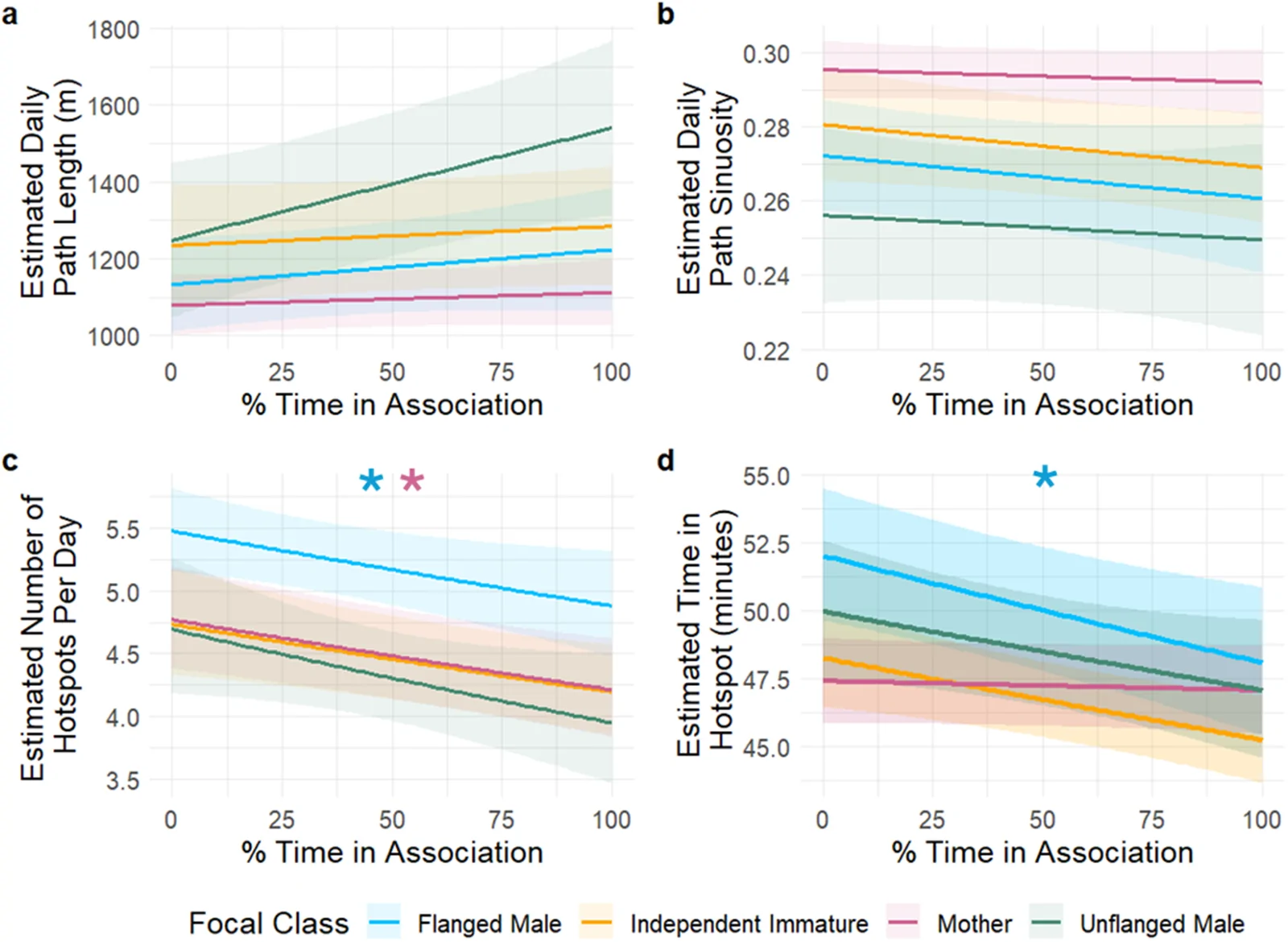
Effects of associations on different spatial metrics of Sumatran orangutans, at the within-class level. Panels show the relationship between the percentage of time spent in association per day and (**a**) daily path length, (**b**) daily path sinuosity, (**c**) number of feeding hotspots visited per day, and (**d**) time spent feeding in a hotspot. A value of 0% time in association represents days when the focal individual was alone. Coloured lines represent each age-sex class, with separate slopes for each. Asterisks represent a significant effect of % Time in association (*p* < 0.05) on the corresponding response metrics, coloured per age-sex class. Lines are derived from model-estimated values with all predictors, except time in association, held at their mean (estimated using the emmeans() function in R), and shaded ribbons indicate 95% confidence intervals

Daily path length: Within classes, individuals did not consistently increase their daily travel distance when spending more time in association (Fig. 5.*a*). Unflanged males showed a positive association between time spent in association and DPL (χ²(1) = 4.95, *p* = 0.026), although the full model was only marginally supported over the null model (χ²(3) = 7.69, *p* = 0.053). Full models were not supported over their corresponding null models for flanged males, mothers, or independent immatures. Fruit availability and rainfall had no significant effects on DPL.

Sinuosity of the daily path: Within age-sex classes, path shapes were generally unaffected by time spent in association (Fig. 5.*b*). The full model explained significantly more variance in daily path sinuosity than it’s corresponding null model for unflanged males (χ²(4) = 14.30, *p* = 0.006), flanged males (χ²(4) = 18.16, *p* = 0.001), and mothers (χ²(4) = 28.73, *p* < 0.001), but not for independent immatures. Time spent in association did not significantly affect sinuosity in any class; however, sinuosity was positively associated with the number of waypoints (unflanged males: χ²(1) = 7.74, *p* = 0.005; flanged males: χ²(1) = 15.19, *p* < 0.001; mothers: χ²(1) = 25.36, *p* < 0.001). Rainfall reduced path sinuosity for unflanged males (χ²(1) = 4.51, *p* = 0.034). Fruit availability had a positive effect on sinuosity for flanged males (χ²(1) = 6.06, *p* = 0.014).

Number of feeding hotspots visited per day: The effect of time spent in association on the number of feeding hotspots visited varied among age-sex classes (Fig. 5.*c*). The full model provided a significantly better fit for the number of feeding hotspots visited per day than the corresponding null model for flanged males (χ²(4) = 18.04, *p* = 0.001) and mothers (χ²(4) = 19.76, *p* < 0.001), but not for unflanged males or immatures. For both flanged males and mothers, time spent in association negatively affected the number of hotspots (flanged males: χ²(1) = 9.67, *p* = 0.002; mothers: χ²(1) = 9.67, *p* = 0.002), and active period duration had a positive effect on the number of hotspots (flanged males; χ²(1) = 9.97, *p* = 0.002, mothers; χ²(1) = 11.09, *p* < 0.001). Fruit availability and rainfall did not significantly affect hotspot visitation.

Time spent feeding in a hotspot: Flanged males spent less time feeding in a hotspot when associating with others, whereas no significant effects of association were detected for the other classes (Fig. 5.*d*). The full model was supported over the null model for flanged males (χ²(4) = 13.81, *p* = 0.008) and marginally so for independent immatures (χ²(4) = 9.46, *p* = 0.051), but not for unflanged males or mothers. Time spent in association at the hotspot negatively affected time spent in a hotspot for (flanged males; χ²(1) = 9.16, *p* = 0.002, and immatures; χ²(1) = 8.02, *p* = 0.005). Active period duration, fruit availability, and rainfall had no significant effects.

### Effects of focal-associate class combination on movement and foraging patterns

**Fig. 6:**
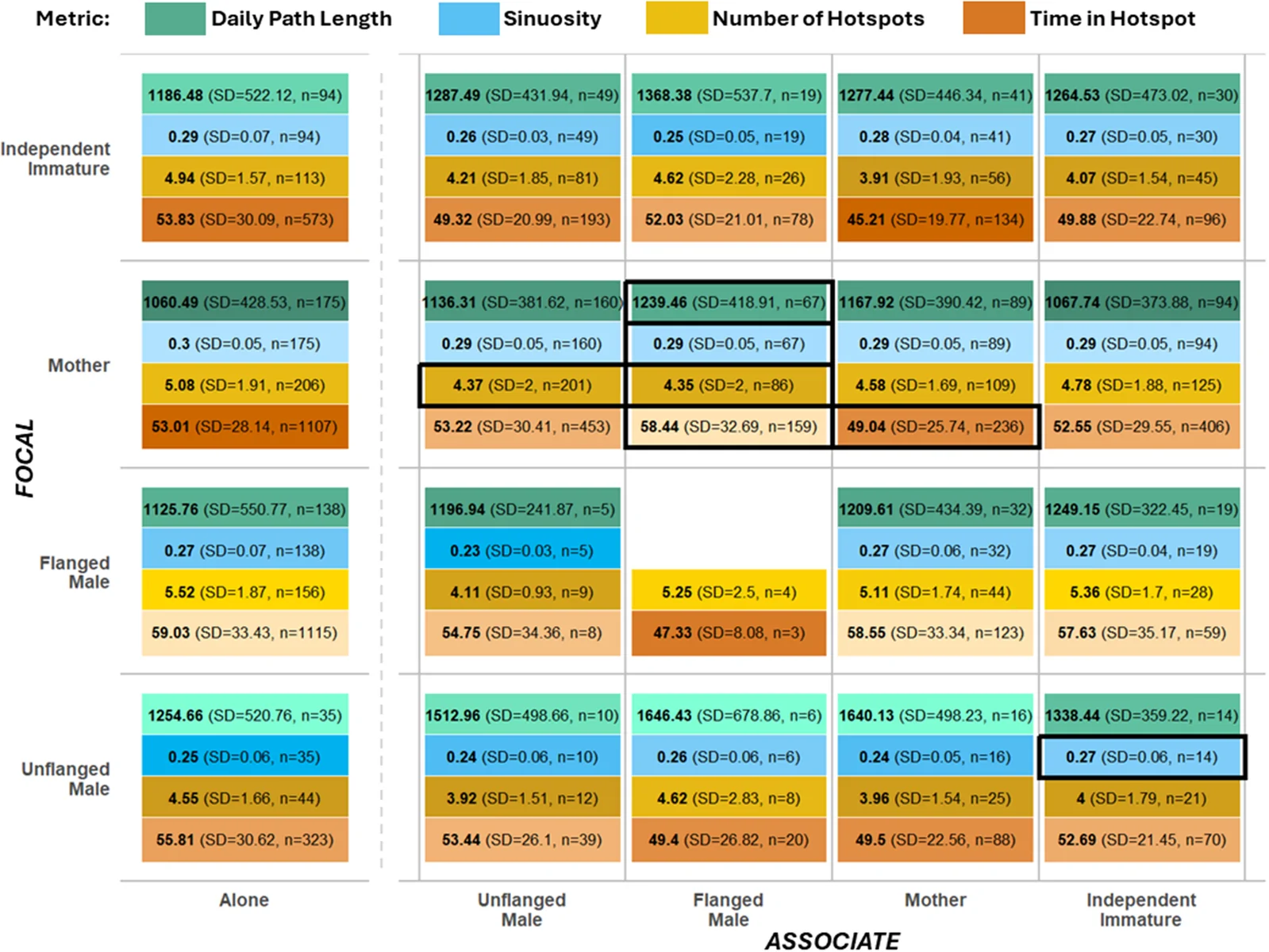
Dyadic spatial metrics by focal and associate age-sex class. Each row represents a focal age-sex class, and each column represents either no associate (alone) or the age-sex class of the associate. Within each cell, four stacked tiles show the average value of metrics: daily path length (DPL), sinuosity, number of hotspots visited per day (No. of HS), and time spent in a hotspot (Time in HS). Tile colour indicates the relative observed value of each metric across each category of associate or when alone, from darkest (lowest) to lightest (highest), with blank cells denoting metrics with no data. Black boxes depict significant effects of the presence of the indicated associate class on the movement metric. n = number of observations per metric

Daily Path Length: At the focal-associate class combination level, mothers were the only focal class whose daily travel distance varied with associate class (Fig. 6). The full models explained significantly more variance in DPL than the corresponding null models for mothers (χ²(6) = 14.67, *p* = 0.023), but not for immatures, unflanged males or flanged males. For mothers, the presence of flanged males (χ²(1) = 7.73, *p* = 0.005) positively affected DPL, while the presence of other classes did not. Fruit availability and rainfall had no significant effect.

Sinuosity of the daily path: Different classes of association partners had generally limited effects on path sinuosity (Fig. 6). The full models explained significantly more variance in path sinuosity compared to the null models for unflanged males (χ²(7) = 17.06, *p* = 0.017), flanged males (χ²(6) = 13.93, *p* = 0.030), and mothers (χ²(7) = 38.71, *p* < 0.001), but not for immatures. For unflanged males, trajectories became more sinuous in the presence of immatures (χ²(1) = 5.58, *p* = 0.018), and mothers’ trajectories became less sinuous in the presence of flanged males (χ²(1) = 4.01, *p* = 0.045). Other predictors also contributed to variation in sinuosity, including number of waypoints in unflanged males, flanged males, and mothers, rainfall in unflanged males, and fruit availability for mothers. Complete model results are provided in Supplementary Table 3.

Number of feeding hotspots visited per day: Associate class affected hotspot visitation primarily for mothers (Fig. 6). For mothers, the full model provided a significantly better fit for the number of feeding hotspots visited per day than the corresponding null model (χ²(7) = 27.35, *p* < 0.001). Mothers visited fewer hotspots when unflanged males (χ²(1) = 9.23, *p* = 0.002) or flanged males (χ²(1) = 4.06, *p* = 0.044) were present, while mothers visited a greater number of hotspots as follow duration increased (χ²(1) = 15.21, *p* < 0.001). Other predictors were non-significant. The full models for hotspot use were not supported over the null models for unflanged males, flanged males, or immatures.

Time spent feeding in a hotspot: Mothers adjusted how long they stayed at a feeding hotspot depending on the class of the associate (Fig. 6). For mothers, the full model explained significantly more variance in the time spent feeding in a hotspot than the null model (χ²(7) = 19.95, *p* = 0.006). The presence of flanged males increased mothers’ time in a hotspot (χ²(1) = 12.01, *p* < 0.001), whereas the presence of other mothers decreased it (χ²(1) = 4.49, *p* = 0.034). Other predictors had no significant effects. No significant effects of associate class were detected for independent immatures, unflanged males, or flanged males.

## Discussion

By integrating fine-scale movement data with detailed behavioural observations, we move beyond describing *how* orangutans travel, to identifying potential reasons *why* they make these movement decisions. This study provided a multidimensional view of how orangutans dynamically adjust movement in response to their immediate social environment and, in doing so, sheds novel light on the potential costs and benefits of sociality. This socially responsive movement supports predictions that this semi-solitary ape experiences measurable ranging and foraging trade-offs when associating with conspecifics, and also that these trade-offs vary across age-sex classes.

Previous research shows that orangutan associations vary across age-sex class [5, 17], reproductive state [18], and habitat productivity [10, 19]. In line with this, our analyses identified the immediate social environment as a consistent driver of movement decisions, with effects varying between focal-associate class combinations, and some limited support for effects of habitat productivity. Because our study is observational, all reported patterns should be interpreted as correlational evidence rather than direct evidence of causation. However, by testing theory-informed predictions using fine-scale behavioural and movement data, we provide evidence for patterns consistent with particular social benefits and costs, while recognising that the benefits themselves were not directly measured.

### Effects of associations on daily path length

We predicted that time spent in association increases daily path length due to coordination demands and social interactions, whereas solitary conditions favour more direct and energy-efficient movement. Our results partially supported this prediction, showing that sociality can impose a measurable energetic cost for orangutans: individuals travelled farther on days with greater social engagement (population-level analyses), but this effect was not consistent across all age-sex classes and social contexts.

Unflanged males generally travelled the farthest, followed by immatures, flanged males, and then mothers. For unflanged males, long paths likely reflect mating strategies involving active tracking and monitoring of potential mates. Moreover, unflanged males typically have less familiarity with the area, and must therefore expand their knowledge of the distribution of resources and conspecifics through exploration after dispersing out of their natal range to avoid inbreeding [7]. Longer travel distances may thus reflect differences in habitat use or ranging strategies that are not directly related to social behaviour, such as searching for specific food resources. Similarly, long paths for immatures are consistent with the exploratory ranging behaviour of dispersing immatures, who must gain spatial knowledge of resource distribution during the development of ecological competence [33, 67], and home range establishment [33].

Contrary to our prediction that less knowledgeable age–sex classes would benefit from the spatial knowledge of associates, we found no effect of associations on daily path length in either unflanged males or immatures. However, the absence of such an effect does not necessarily indicate that immatures or unflanged males do not use others’ spatial knowledge to inform movement decisions. Social information could instead influence other aspects of movement efficiency, such as resource encounter rates, route selection, or the ability to locate profitable feeding sites, which are not captured by travel distance alone, but may be captured in the other measured metrics. For immatures it is also possible that the influence of associates varies across age and was therefore not captured in the present model. Longitudinal or cross-sectional analyses would help determine whether immatures benefit from associations at specific ages.

The finding that mothers and flanged males tended to travel shorter daily paths than the other two classes is consistent with their need to balance energy expenditure against foraging gains and is likely facilitated by their greater knowledge of resource locations resulting from their stable home ranges. Mothers, in particular, incur the high energetic costs of pregnancy and offspring care, whereas flanged males bear the energetic costs associated with their large body size, leading both classes to prioritize energy conservation.

Importantly, at the population level, increased daily path length reflected longer periods of activity rather than faster movement. This indicates that individuals in social contexts do not necessarily move more intensively, but instead remain active for longer periods, resulting in greater total daily travel distances. Given the high energetic costs of locomotion in orangutans, driven by their large body and brain size as well as arboreal lifestyle, even modest increases in daily travel may create significant energetic costs [68].

Results at the focal-associate class combination level further highlighted that the effects of social context are partner-specific. Against our prediction, mothers did not increase their daily path length when in the presence of unflanged males but did so when in the presence of flanged males. Females are generally thought to preferentially associate with flanged males as mating partners, while tending to avoid unflanged males [18, 28], which often engage in coercive mating attempts. Thus, longer paths when associating with flanged males could potentially reflect female mate-seeking behaviour driven by female choice. However, the finding that flanged males were more often following females, as shown by the movement order analysis, does not support this interpretation. Notably, females led travel when with both flanged and more pronouncedly with unflanged males. Flanged males that are newly immigrated or only recently attained dominance likely have a lower probability of paternity and could therefore represent a potential threat to infants [69], similar to unflanged males. Our results may thus reflect females’ prioritization of infant protection and their active attempts to maintain control over travel direction or spatial positioning relative to potentially coercive males. Subsequently, associations with either class of males can impose costs on females, reflecting a form of sexual conflict, with variation depending on male morph and context, aligning with published research on costs of associating with males [15]. In this light, our findings suggest that mothers adjust their movement not simply in response to associate presence per se, but according to the perceived risks and benefits associated with specific males.

The absence of focal-associate class combination-specific effects in immatures, flanged males, and unflanged males does not necessarily indicate that the influence of associates on path length are absent. Instead, such effects may be expressed specifically during periods of coordinated travel. Across the full nest-to-nest active period, other activities, such as resting, feeding, or socialising with an associate, may have little influence on overall path length or its structure. Though this study could not isolate periods of coordinated travel, future analyses could help establish whether associate-related effects on path length are context-dependent.

Against our prediction, unflanged males did not show increased travel distance when with (generally more dominant) flanged males. However, activity budget behavioural data revealed that when unflanged and flanged males associated with other flanged males, they had the highest proportions of time spent travelling, and the greatest reduction in time spent feeding, compared to other dyads, providing further evidence for avoidance and dominance-related displacement, and subsequent energetic costs. Moreover, there were no data available on the presence of flanged males with other flanged males due to the lack of full-day nest-to-nest observations with sufficient GPS data, which provides further evidence for avoidance behaviour between the dominant males.

### Effects of associations on daily path sinuosity

We predicted that increased time spent in association would increase path sinuosity due to increased opportunities to coordinate movement decisions, such as travelling together to a shared goal. This prediction was not supported at the population level: instead, there was a trend towards straighter paths when with associates, though this effect was not significant. Results differed at the within-class and the focal-associate class combination levels. This suggests that while social factors such as feeding competition or the opportunities to benefit from another’s spatial knowledge do not influence the directness of travel routes, class-specific energetic requirements and nutritional needs may.

At the population level, mothers exhibited more sinuous paths than unflanged males, which most likely reflects contrasting ranging strategies. Mothers may adopt more sinuous movement patterns as they forage intensively within localised areas, repeatedly revisiting resource patches to meet the high energetic demands, and potential nutrient requirements of pregnancy, lactation [70–72] and infant care, while minimising travel costs. Mothers may also deviate from spatially efficient travel paths, as they must select routes with smaller canopy gaps that allow their dependent offspring to cross [73], and occasionally return to retrieve them if they fall behind.

In contrast to the expectation that unflanged males may exhibit less efficient travel due to more limited spatial knowledge, we observed a trend towards longer but straighter travel paths in unflanged males compared with flanged males, although this pairwise comparison was not statistically significant. Such straighter trajectories could indicate a strategy that enhances encounter rates with females while improving travel efficiency, thereby offsetting the energetic costs of their long daily paths.

Overall, we found limited evidence for focal-associate class combination effects on path sinuosity within the different focal classes. Most associations did not result in consistent changes in sinuosity compared with solitary movement, suggesting that orangutans generally show limited modification of path shape when travelling with others. However, some focal-associate class combination effects on sinuosity were evident. For example, increased sinuosity in unflanged males in the presence of immatures, which may reflect adjustments in movement to accommodate less experienced partners, and reduced sinuosity for mothers when accompanied by flanged males, potentially indicating attempts to avoid coercion attempts (see above). Moreover, individuals may have independent yet aligned travel goals with associates, such as in areas of high resource abundance, therefore, the observed coordination between individuals’ movements may reflect shared environmental preferences rather than active adjustment of path shape in response to a conspecific’s movements. Taken together, these results suggest that path sinuosity is primarily structured by focal age-sex class, reflecting differences in energetic requirements and ranging strategies, but also that the immediate social environment can shape movement patterns in specific contexts.

### Effects of associations on feeding hotspot use

We predicted that sociality would increase the number of feeding hotspots visited due to scramble competition, and reduce the time spent at each hotspot because of resource depletion. Our results partially supported this prediction: at the population level, associations reduced hotspot residence time, consistent with increased depletion, but contrary to expectations, individuals visited fewer rather than more hotspots when spending more time with others. These findings provide detailed insight into feeding competition, suggesting that individuals do not compensate for visiting fewer hotspots by prolonging their visits. Instead, sociality appears to constrain both the breadth and intensity of resource exploitation. However, this pattern may also partly reflect a methodological effect driven by patch depletion rates. When individuals are in association, increased feeding competition may lead to faster resource depletion, resulting in shorter visits that fall below the threshold required for a location to be classified as a hotspot. In contrast, when alone, individuals may exploit the same patches for longer due to lower depletion rates, increasing the likelihood that these locations are identified as hotspots. Thus, some patches with high resource potential may be underrepresented as hotspots in social contexts, potentially exemplified by our result of fewer hotspots visited and less time spent in them when in longer associations. To explore whether social effects on resource depletion influenced hotspot identification, we conducted an additional analysis using a socially weighted hotspot definition that incorporated the number of individuals present in a feeding patch (Supplementary method 1). The weighted analysis produced the same overall patterns as the unweighted measure, suggesting that our main conclusions were robust to this alternative hotspot definition.

Age-sex class differences in feeding hotspot use were also evident; flanged males exploited the greatest number of feeding hotspots, and also spent more time in them, than the other age-sex classes. This pattern may reflect greater local knowledge, and a strategy of exploiting and exhausting foraging resources without the added costs of searching for alternative resources, given the high energetic costs of locomotion in flanged males [36, 72, 74]. The social dominance of flanged males also likely allows them to monopolise resources and therefore displace associates. However, despite exploiting hotspots the most, as the percentage of time spent in association increased, they visited fewer hotspots, and spent less time in them (within-class level analyses). Similar effects were seen for mothers, who visited fewer hotspots as association time increased, and immatures, whose time spent in a hotspot also decreased when spending longer time in associations.

We found several focal-associate class combination level trends suggesting that the class of associates may differentially influence levels of feeding competition and evidence for individuals’ strategies to compensate for it (Focal-associate class combination level analyses). For instance, mothers visited fewer hotspots when around flanged and unflanged males, but extended hotspot residence time in the presence of flanged males. This result suggests that mothers compensate for the reduced number of hotspots visited when with flanged males by exploiting them more thoroughly, consistent with energy conservation strategies. This result may also reflect variation in the nature of female–flanged male associations. Although females may experience costs associated with coercive interactions with some flanged males, other flanged males may be more socially tolerant, and females may actively associate with these individuals when potential benefits outweigh costs [18, 27] whereas they tend to avoid unflanged males who actively seek and often perturb them [75]. Thus, the effects of flanged male presence on female movement and resource use are likely to depend on the identity and social history of the individuals involved. Orangutan mothers are known to display high levels of social tolerance to other females and immatures, especially their close relatives [76, 77]. However, the results of this study reveal that the presence of mothers reduces time in a hotspot for other mothers. This highlights that competition and resource depletion are likely the key drivers of foraging patch exploitation, when mating benefits are absent.

Examining focal-associate class combinations revealed that the class of social partners, plays a critical role in shaping foraging behaviour for some age-sex classes, particularly mothers. In contrast, adult males, particularly flanged males, showed relatively limited sensitivity to the age-sex class of associates, suggesting greater autonomy in their movement and foraging decisions. For mothers, this pattern may reflect greater social tolerance, potentially because associations with a wider range of individuals may provide benefits in environments where resource locations and social opportunities are uncertain, therefore, social flexibility may itself be an important component of movement and foraging strategies.

### Social modulation of activity budgets and movement initiation

We predicted that associations would alter activity budgets and movement initiation depending on the costs and benefits associated with different social partners. Overall, this prediction was partially supported; activity budget patterns were broadly conserved across social contexts, except for the time individuals allocated to movement and social behaviour, as well the frequency of initiating travel. Across all contexts, whether alone or with different classes of associates, individuals devoted the largest proportion of their time to feeding (around 50–60%), followed by movement and resting. Nevertheless, social associations may influence the functional context in which these activities occur, by adding opportunities for information transfer. Feeding in close proximity to associates may be particularly important for naïve individuals, such as immatures and unflanged males, by facilitating the acquisition of food locating, and extractive foraging skills. Travel with partners may enhance spatial learning of resource distribution, while resting alongside associates may provide more opportunities for observational social learning.

Although overall activity budgets remained broadly similar, associations generally resulted in a greater proportion of time spent moving, longer total active periods, and consequently increased path length, all of which carry energetic costs. However, individuals did not appear to offset these costs by increasing energy intake by spending more time feeding; instead contrasting effects were observed, most notably seen in flanged males who faced the most substantial reduction in time spent feeding when associating with unflanged males, with a modest increase in time allocated to resting, socialising, and other activities (such as independent exploration, and long-call vocalisations) instead. Across all age-sex classes, associations with flanged males were also associated with increased resting time, likely reflecting the characteristically higher resting requirements of flanged males due to the energetic constraints of their larger body size.

Movement bout order adds another layer of evidence to what factors underlie movement decisions. Following an associate can create opportunities for social learning, particularly from more knowledgeable individuals, whereas leading the movement suggests active spatial decision making. Leading a movement bout may reflect either a preference to navigate toward a goal regardless of an associate’s presence, or avoidance of the associate, which could impose additional energetic costs by causing deviations from a goal-directed path.

Patterns of dyadic movement order were consistent with female mate choice and the contrasting mating strategies of the two male morphs; unflanged males tended to follow mothers, and to a lesser extent immatures, consistent with their strategy of seeking mates. Consistent with previous observations, females associate preferentially with flanged males [18, 28], and avoid coercive unflanged males, as indicated by a higher proportion of leading movement bouts, reflecting their mating preference. Flanged males often displaced other males, a pattern reflected in the proportions of movement bouts and the low number of these dyadic associations in general.

Independent immatures were the most social class, with an associate more often than they were alone, though engaged in relatively little direct social interaction, compared to the other age-sex classes. Despite limited direct interactions, their greater level of sociality may confer indirect social benefits: by being within sight of more experienced individuals, they could gain access to information about resource locations, conspecific locations, or predator avoidance without the costs of direct interaction or conflict. In this way, social association may act as a low-risk learning environment, in which immatures have the opportunity to benefit from the knowledge and experience of others. Our results also showed that immatures followed tolerant or knowledgeable resident individuals [78], especially their own mothers [16], but seemed to avoid potentially risky males, indicating selective social use of information. This combination supports published literature stating an exploratory [14, 33, 79], learning-oriented [67] movement strategy.

Conversely, flanged males were the least social class, though when with an associate, they did invest more time in direct social behaviour, particularly mating and observing mothers, and displaying aggression to other males, resulting in more active social behaviour for a mating or dominance goal, rather than passive social behaviour when feeding or travelling. Their large body size and social dominance (reflected by leading movement more than following) suggest priority access to resources and reduced need to modify travel due to associates, aligning with an energy-conserving, dominance-based movement strategy.

Mothers had a more stable activity budget regardless of associate class, and they frequently led movement, likely avoiding costly associations [5]. They were sensitive to associate age-sex class, reflected in their longer daily paths, and use of fewer foraging hotspots when with flanged males, but varied time spent in a hotspot depending on associate class. Together, these patterns indicate an energetic conservation and infant-protection-driven strategy shaped by the demands of caregiving.

Unflanged males varied in their allocation of time to social behaviours depending on the age-sex class of the associate, likely due to seeking mates and avoiding rival males. Interestingly, unflanged males and independent immature dyads devoted the highest proportion of time in association to feeding, which may help these classes, who have relatively limited knowledge of resource locations, share information about where to find food.

### Ecological effects on movement and foraging behaviour

Interestingly, ecological variation, specifically fruit availability, did not have a consistent influence on movement, despite the largest proportion of the active period devoted to foraging and consuming food. Only some age-sex-class-specific ecological effects emerged at the within-class level. Unflanged males travelled along less sinuous paths during periods of increased rainfall, suggesting more direct movement under challenging environmental conditions, whereas flanged males showed greater path sinuosity during periods of high local fruit availability, consistent with more localised searching or patch use when resources were abundant. This absence of consistent effects of fruit availability may be specific to the Suaq Balimbing site, which benefits from relatively high and stable food availability, compared to other orangutan research sites. Similar observations have been reported in other studies at Suaq, such as when alternative measures of fruit availability (eFAI; which calculated the actual fruit availability experienced by the individual, rather than habitat-level availability) showed minimal effects on ranging [80]. A different study on the same population found that fruit availability did not show any impact on day journey length [49], whereas another found contrasting results on male path length [81]. Moreover, additional studies found that fruit availability at Suaq Balimbing was not strongly linked to party size [5], insect-feeding preferences [82], or social play [25], in contrast to other sites where stronger effects have been observed. Hence, the effects of fruit availability are likely to be less pronounced at Suaq than other sites.

## Limitations

Several limitations should be considered. Because our study was observational and did not directly measure the benefits that individuals may derive from associations, interpretations concerning the benefits of sociality remain hypotheses supported by predicted behavioural patterns rather than directly demonstrated benefits.

The number of GPS waypoints strongly influenced sinuosity, so we included it as a control covariate because it correlates with both active period duration and data resolution. While active period duration limits total movement, resolution directly affects calculated metrics by capturing more fine-scale path deviations, making this control essential to distinguish biological from methodological effects. However, the nature of the effect of the number of waypoints on sinuosity would need to be explored in detail to assure that adding it as a linear control is the best practice. Importantly, the observed age-sex differences in movement remained robust after accounting for waypoint number, indicating that our main conclusions are driven by genuine behavioural differences rather than artefacts of active period duration or GPS sampling density.

Furthermore, analyses on the focal-associate class combination level varied in sample size, particularly for males, given that data are collected opportunistically, whereby low sample size itself may provide evidence for social intolerance, but also reduced power to detect subtle social effects. The absence of significant effects in the current dataset therefore does not fully rule out their presence. Conversely, because of sample size limitations, we did not control for repeated testing, for example by adjusting our significance threshold [83]. While it is common practice for studies with sample size limitations to not adjust significance thresholds to avoid reducing statistical power, it does inflate the risk of false positive effects.

Finally, fruit availability, though a method consistent within published literature, is measured at relatively coarse temporal scales, which do not accurately reflect fine-scale resource depletion, or orangutans’ dietary preference. Future work integrating high-resolution resource mapping may reveal additional ecological influences not captured in this study.

## Conclusion

By using orangutans as a model system and leveraging an exceptionally detailed long-term dataset on movement and behaviour, we generated broader insights into the energetic costs of social associations linked to movement and foraging, and how potential benefits of associations can offset them. As semi-solitary apes that, due to their large body size and arboreal lifestyle, face pronounced energetic constraints [72], orangutans provide a valuable framework to investigate the costs and benefits of social association [10, 13, 84]. Our study concludes that orangutan movement is best understood as age-sex-class-driven and individually expressed, yet socially shaped in many aspects. Social associations may influence when and how far individuals travel, and how they utilize foraging hotspots, but they do not appear to strongly restructure the fine-scale geometry of daily movement paths. These findings suggest that the energetic costs of sociality arise primarily through changes in movement effort and resource use associated with feeding competition, rather than through fundamental changes in path efficiency. At the same time, the context-dependent nature of these behavioural adjustments indicates that individuals may tolerate the costs of social travel when associations provide sufficient benefits to offset these costs. This study sheds novel light on the behavioural mechanisms underlying feeding competition as well as how these costs of associations are dynamically weighted by the benefits of spatial knowledge sharing, such as skill acquisition [14, 39], enhanced discovery of resources [41], protection from harassment [5], and mating opportunities [12, 23].

More broadly, these results contribute to the growing evidence that even species traditionally characterised by their semi-solitary nature exhibit socially responsive movement strategies, akin to those observed in more gregarious species, challenging simple dichotomies between solitary and group-living species. By integrating spatial metrics with fine-scale behavioural data, such as activity budgets and order of movement bouts, we were able to move beyond describing *how* individuals move, to provide possible interpretations on *why*, based on how the observed patterns matched our predictions.

Taken together, our study emphasises that trade-offs between costs and benefits of associations are flexible and context-dependent, and should be considered central to understanding ranging behaviour. Recognising this socially mediated flexibility is particularly important for conservation, as changes in population density particularly in fragmented habitats may alter these trade-offs and, consequently, the energetic balance of individuals.

## Supplementary Information

Below is the link to the electronic supplementary material.

Supplementary Material 1

Supplementary Material 2

Supplementary Material 3

Supplementary Material 4

Supplementary Material 5

Supplementary Material 6

Supplementary Material 7

Supplementary Material 8

## Data Availability

The datasets used and/or analysed during the current study are available from the corresponding author on reasonable request.

## Abbreviations

DPL: Daily Path Length
LMMs: Linear mixed models
GLMMs: Generalised linear mixed models
LRTs: Likelihood ratio tests
HS: Hotspot
ANOVA: Analysis of variance
i.e.: Id est
n: Number
No.H: Number of hotspots
m: Metres
χ²: Chi-squared statistic
CTSD: Continuous-time speed and distance
CTMM: Continuous time movement model
