# Supplementary material for: The immediate social environment dynamically shapes movement and foraging in wild Sumatran orangutans (Pongo abelii)
Source: Mov Ecol. 2026 Oct 21;14:74. doi: 10.1186/s40462-026-00713-2 (PMC13689139; doi:10.1186/s40462-026-00713-2)
Supplement: Supplementary file 1 [file 40462_2026_713_MOESM1_ESM.docx]

***Supplementary table 1.*** ***Sensitivity analysis of GPS completeness threshold on movement models.***

| *Movement Metric* | *Completeness Threshold* | | *Sample Size* | *Focal individuals* | *Estimate Time In Association* | *SE* | *t value* |
| --- | --- | --- | --- | --- | --- | --- | --- |
| Daily path length | | ≥25% | 1169 | 85 | 63.5400 | 32.3600 | 1.96 |
| Daily path length | | ≥50% | 1139 | 82 | 63.5400 | 32.3600 | 1.96 |
| Daily path length | | ≥75% | 981 | 72 | 63.5400 | 32.3600 | 1.96 |
| Daily path length | | 100% | 437 | 52 | 81.1700 | 46.9700 | 1.73 |
| Path sinuosity | | ≥25% | 1169 | 85 | -0.0097 | 0.0036 | -2.70 |
| Path sinuosity | | ≥50% | 1139 | 82 | -0.0095 | 0.0036 | -2.63 |
| Path sinuosity | | ≥75% | 981 | 72 | -0.0073 | 0.0039 | -1.86 |
| Path sinuosity | | 100% | 437 | 52 | -0.0058 | 0.0059 | -1.00 |

Missing GPS locations can influence movement estimates, particularly metrics based on path geometry, such as sinuosity. To evaluate which GPS completeness threshold to use in the main analyses, we repeated the daily path length and path sinuosity models using alternative GPS completeness criteria (≥25%, ≥50%, ≥75%, and 100%). For each threshold, we refitted the models using the same model structures as the main analyses (Model1_DPL and Model1_Sin*; see Supplementary table 2*). Effects remained consistent across thresholds.

Increasing the completeness threshold reduced the available sample size. For daily path length analyses, the dataset decreased from 1,169 follow-days involving 85 focal individuals at the ≥25% threshold to 981 follow-days involving 72 focal individuals at the ≥75% threshold, representing a loss of 188 follow-days (16.1%) and 13 focal individuals (15.3%). Requiring complete GPS data (100% completeness) resulted in a substantially greater reduction, leaving 437 follow-days from 52 focal individuals, corresponding to losses of 732 follow-days (62.6%) and 33 focal individuals (38.8%) relative to the ≥25% threshold.

Despite reductions in sample size, the estimated effect of time spent in association on daily path length was highly consistent across completeness thresholds. The estimated increase in daily path length associated with time spent in association was 63.5 m at the ≥25%, ≥50%, and ≥75% thresholds. The 100% completeness threshold produced a slightly larger estimate (81.2 m), but this was accompanied by increased uncertainty, likely reflecting the substantially reduced sample size (*Supplementary table 1*).

For path sinuosity, the estimated relationship between time spent in association and sinuosity remained negative across all completeness thresholds. However, the strength of this association decreased with increasingly strict completeness criteria, and uncertainty increased as sample sizes declined, particularly under the 100% completeness threshold (*Supplementary table 1*).

Together, these sensitivity analyses indicate that the ≥75% GPS completeness threshold provided a suitable balance between reducing potential bias from incomplete movement trajectories and retaining sufficient sampling effort and individual representation for robust statistical inference.
