# Supplementary material for: The immediate social environment dynamically shapes movement and foraging in wild Sumatran orangutans (Pongo abelii)
Source: Mov Ecol. 2026 Oct 21;14:74. doi: 10.1186/s40462-026-00713-2 (PMC13689139; doi:10.1186/s40462-026-00713-2)
Supplement: Supplementary file 2 [file 40462_2026_713_MOESM2_ESM.docx]

***Supplementary table 2. Model structures and sample sizes.*** *Statistically significant results are shown in bold.*

| **Model number** | **Dataset** | **Model** | **Sample size** |
| --- | --- | --- | --- |
| **LEVEL 1** | | | |
| **Effects of Age Sex Class** | | | |
| *Model1_DPL* | **All individuals** | lmer(DPL ~ **FocalAgeSexClass** + **TimeInAssociation** + FruitAvailability + **TotalWaypointsPerPath** + Rainfall + (1\|focalID)) | *992* |
| *Model1_Sin* | **All individuals** | lmer(Sinuosity ~ **FocalAgeSexClass** + TimeInParty + FruitAvailability + **TotalWaypointsPerPath** + Rainfall + (1\|focalID)) | *992* |
| *Model1_No.H* | **All individuals** | glmmTMB(NoOfHotspotsPerFollow ~ **FocalAgeSexClass** + **TimeInAssociation** + FruitAvailability + **ActivePeriodDuration** + Rainfall + (1\|focalID), family = compois() ) | *992* |
| *Model1_Time* | **All individuals** | lmer(log(TimeInHotspots) ~ **FocalAgeSexClass** + **TimeInAssociationAtHotspot** + FruitAvailability + ActivePeriodDuration + Rainfall + (1\|focalID) + (1\|followID)) | *6571* |
| **LEVEL 2** | | | |
| **DPL ~ Time in Association (per focal age-sex class)** | | | |
| *Model2_DPL_UF* | **Unflanged Males** | lmer(DPL ~ **TimeInAssociation** + FruitAvailability + TotalWaypointsPerPath + Rainfall + (1\|focalID) ) | *64* |
| *Model2_DPL_FL* | **Flanged Males** | lmer(DPL ~ TimeInAssociation + FruitAvailability + **TotalWaypointsPerPath** + Rainfall + (1\|focalID) ) | *207* |
| *Model2_DPL_M* | **Mothers** | lmer(DPL ~ TimeInAssociation + FruitAvailability + **TotalWaypointsPerPath** + Rainfall + (1\|focalID) ) | *508* |
| *Model2_DPL_I* | **Immatures** | lmer(DPL ~ TimeInAssociation + FruitAvailability + **TotalWaypointsPerPath** + Rainfall + (1\|focalID) ) | *213* |
| **Sinuosity ~ Time in Association (Per Focal Class)** | | | |
| *Model2_Sin_UF* | **Unflanged Males** | lmer(Sinuosity ~ TimeInAssociation + FruitAvailability + **TotalWaypointsPerPath** + **Rainfall** + (1\|focalID) ) | *64* |
| *Model2_Sin_FL* | **Flanged Males** | lmer(Sinuosity ~ TimeInAssociation + FruitAvailability + **TotalWaypointsPerPath** + Rainfall + (1\|focalID) ) | *207* |
| *Model2_Sin_M* | **Mothers** | lmer(Sinuosity ~ TimeInAssociation + **FruitAvailability** + **TotalWaypointsPerPath** + Rainfall + (1\|focalID) ) | *508* |
| *Model2_Sin_I* | **Immatures** | lmer(Sinuosity ~ TimeInAssociation + FruitAvailability + TotalWaypointsPerPath + Rainfall + (1\|focalID) ) | *213* |
| **No Of Hotspots ~ Time in Association (per focal age-sex class)** | | | |
| *Model2_No.H_UF* | **Unflanged Males** | glmmTMB(NoOfHotspotsPerFollow ~ TimeInAssociation + FruitAvailability + ActivePeriodDuration + Rainfall + (1\|focalID), family = compois() ) | *89* |
| *Model2_No.H_FL* | **Flanged Males** | glmmTMB(NoOfHotspotsPerFollow ~ **TimeInAssociation** + FruitAvailability + **ActivePeriodDuration** + Rainfall + (1\|focalID), family = compois() ) | *250* |
| *Model2_No.H_M* | **Mothers** | glmmTMB(NoOfHotspotsPerFollow ~ **TimeInAssociation** + FruitAvailability + **ActivePeriodDuration** + Rainfall + (1\|focalID), family = compois() ) | *615* |
| *Model2_No.H_I* | **Immatures** | glmmTMB(NoOfHotspotsPerFollow ~ TimeInAssociation + FruitAvailability + ActivePeriodDuration + Rainfall + (1\|focalID), family = compois() ) | *281* |
| **Time In Hotspots ~ Time In Association (per focal age-sex class)** | | | |
| *Model2_Time_UF* | **Unflanged Males** | lmer(log(TimeInHotspots) ~ TimeInAssociationAtHotspot + FruitAvailability + ActivePeriodDuration + Rainfall + (1\|focalID) + (1\|followID)) | *610* |
| *Model2_Time_FL* | **Flanged Males** | lmer(log(TimeInHotspots) ~ **TimeInAssociationAtHotspot** + FruitAvailability + ActivePeriodDuration + Rainfall + (1\|focalID) + (1\|followID)) | *1664* |
| *Model2_Time_M* | **Mothers** | lmer(log(TimeInHotspots) ~ TimeInAssociationAtHotspot + FruitAvailability + ActivePeriodDuration + Rainfall + (1\|focalID) + (1\|followID)) | *2932* |
| *Model2_Time_I* | **Immatures** | lmer(log(TimeInHotspots) ~ **TimeInAssociationAtHotspot** + FruitAvailability + ActivePeriodDuration + Rainfall + (1\|focalID) + (1\|followID)) | *1365* |
| **LEVEL 3** | | | |
| **DPL ~ Association Dyad (per focal age-sex class)** | | | |
| *Model3_DPL_UF* | **Unflanged Males** | lmer(DPL ~ PresenceUnflanged + PresenceFlanged + PresenceMother + PresenceImmature + FruitAvailability + TotalWaypointsPerPath + Rainfall + (1\|focalID) ) | *28* |
| *Model3_DPL_FL*  *** | **Flanged Males** | lmer(DPL ~ PresenceUnflanged + + PresenceMother + PresenceImmature + FruitAvailability + TotalWaypointsPerPath + Rainfall + (1\|focalID) ) | *65* |
| *Model3_DPL_M* | **Mothers** | lmer(DPL ~ **PresenceUnflanged** + PresenceFlanged + **PresenceMother** + PresenceImmature + FruitAvailability + **TotalWaypointsPerPath** + Rainfall + (1\|focalID) ) | *330* |
| *Model3_DPL_I* | **Immatures** | lmer(DPL ~ **PresenceUnflanged** + PresenceFlanged + PresenceMother + PresenceImmature + FruitAvailability + **TotalWaypointsPerPath** + Rainfall + (1\|focalID) ) | *115* |
| **Sinuosity ~ Association Dyad (per focal age-sex class)** | | | |
| *Model3_Sin_UF* | **Unflanged Males** | lmer(Sinuosity ~ PresenceUnflanged + PresenceFlanged + PresenceMother + **PresenceImmature** + FruitAvailability + **TotalWaypointsPerPath** + **Rainfall** + (1\|focalID) ) | *28* |
| *Model3_Sin_FL*  *** | **Flanged Males** | lmer(Sinuosity ~ PresenceUnflanged + + PresenceMother + PresenceImmature + FruitAvailability + **TotalWaypointsPerPath** + Rainfall + (1\|focalID) ) | *65* |
| *Model3_Sin_M* | **Mothers** | lmer(Sinuosity ~ PresenceUnflanged + PresenceFlanged + PresenceMother + PresenceImmature + FruitAvailability + **TotalWaypointsPerPath** + Rainfall + (1\|focalID) ) | *330* |
| *Model3_Sin_I* | **Immatures** | lmer(Sinuosity ~ PresenceUnflanged + PresenceFlanged + PresenceMother + PresenceImmature + FruitAvailability + TotalWaypointsPerPath + Rainfall + (1\|focalID) ) | *115* |
| **No Of Hotspots ~ Association Dyad (per focal age-sex class)** | | | |
| *Model3_No.H_UF* | **Unflanged Males** | glmmTMB (NoOfHotspotsPerFollow ~ PresenceUnflanged + PresenceFlanged + PresenceMother + PresenceImmature + FruitAvailability + ActivePeriodDuration + Rainfall + (1\|focalID), family = compois()) | *44* |
| *Model3_No.H_FL* | **Flanged Males** | glmmTMB (NoOfHotspotsPerFollow ~ PresenceUnflanged + PresenceFlanged + PresenceMother + PresenceImmature + FruitAvailability + ActivePeriodDuration + Rainfall + (1\|focalID), family = compois()) | *90* |
| *Model3_No.H_M* | **Mothers** | glmmTMB (NoOfHotspotsPerFollow ~ **PresenceUnflanged** + **PresenceFlanged** + PresenceMother + PresenceImmature + FruitAvailability + **ActivePeriodDuration** + Rainfall + (1\|focalID), family = compois()) | *600* |
| *Model3_No.H_I* | **Immatures** | glmmTMB (NoOfHotspotsPerFollow ~ PresenceUnflanged + PresenceFlanged + PresenceMother + PresenceImmature + FruitAvailability + ActivePeriodDuration + Rainfall + (1\|focalID), family = compois()) | *163* |
| **Time In Hotspot ~ Association Dyad (per focal age-sex class)** | | | |
| *Model3_Time_UF* | **Unflanged Males** | lmer(log(TimeInHotspots) ~ PresenceUnflanged + PresenceFlanged + PresenceMother + PresenceImmature + FruitAvailability + ActivePeriodDuration + Rainfall +(1\|focalID) + (1\|followID)) | *170* |
| *Model3_Time_FL* | **Flanged Males** | lmer(log(TimeInHotspots) ~ PresenceUnflanged + PresenceFlanged + PresenceMother + PresenceImmature + FruitAvailability + ActivePeriodDuration + Rainfall + (1\|focalID) + (1\|followID)) | *220* |
| *Model3_Time_M* | **Mothers** | lmer(log(TimeInHotspots) ~ PresenceUnflanged + **PresenceFlanged** + **PresenceMother** + PresenceImmature + FruitAvailability + ActivePeriodDuration + Rainfall + (1\|focalID) + (1\|followID)) | *2849* |
| *Model3_Time_I* | **Immatures** | lmer(log(TimeInHotspots) ~ PresenceUnflanged + PresenceFlanged + **PresenceMother** + PresenceImmature + FruitAvailability + **ActivePeriodDuration** + Rainfall + (1\|focalID) + (1\|followID)) | *491* |

** There were no data available on the presence of flanged males with other flanged males for full-day follows with sufficient GPS data for use in* ***Model3_DPL_FL*** *and* ***Model3_Sin_FL***
