# Supplementary material for: The immediate social environment dynamically shapes movement and foraging in wild Sumatran orangutans (Pongo abelii)
Source: Mov Ecol. 2026 Oct 21;14:74. doi: 10.1186/s40462-026-00713-2 (PMC13689139; doi:10.1186/s40462-026-00713-2)
Supplement: Supplementary file 3 [file 40462_2026_713_MOESM3_ESM.docx]

***Supplementary table 3. Fixed and random effects from linear and generalised linear mixed-effects models investigating predictors of movement behaviour****. Response variables include daily path length, path sinuosity, number of hotspots visited per day, and log-transformed time spent in a hotspot. Fixed effects comprised social association metrics, fruit availability, rainfall, number of GPS waypoints, and active period duration variables. Random intercepts were fitted for focal identity (and follow identity when applicable). Marginal and conditional R² values are reported.*

| **Model** | **Response** | **Predictors** | **Estimates** | **Std. Error** | **Statistic** | ***p*** | **Marginal R^2^ / Conditional R^2^** |
| --- | --- | --- | --- | --- | --- | --- | --- |
| Model_1_DPL | **Daily Path Length** | **Fixed effects** |  |  |  |  |  |
| *n = 971 observations;* |  | (Intercept) | 1033.597 | 74.955 | 13.790 | *< 0.001* | 0.026 / 0.145 |
| *70 individuals* |  | Class [Independent Immature] | 78.071 | 73.159 | 1.067 | *0.286* |  |
|  |  | Class [Mother] | -25.897 | 74.693 | -0.347 | *0.729* |  |
|  |  | Class [Unflanged Male] | 200.289 | 87.658 | 2.285 | ***0.023*** |  |
|  |  | % Time in association | 68.667 | 32.069 | 2.141 | *0.033* |  |
|  |  | **Controls** |  |  |  |  |  |
|  |  | Fruit availability | 10.076 | 5.112 | 1.971 | ***0.049*** |  |
|  |  | Rainfall | -0.554 | 0.960 | -0.577 | ***0.564*** |  |
|  |  | **Random effects** | **Variance** | **SD** |  |  |  |
|  |  | Focal ID (Intercept) | 26746.000 | 163.500 |  |  |  |
|  |  | Residual | 192963.000 | 439.300 |  |  |  |
| Model_1_Sin | **Daily Sinuosity** | **Fixed effects** |  |  |  |  |  |
| *n = 992 observations;* |  | (Intercept) | 0.164 | 0.018 | 8.967 | *<0.001* | 0.091 / 0.150 |
| *72 individuals* |  | Class [Independent Immature] | 0.006 | 0.008 | 0.779 | *0.436* |  |
|  |  | Class [Mother] | 0.021 | 0.008 | 2.727 | *0.011* |  |
|  |  | Class [Unflanged Male] | -0.018 | 0.009 | -1.939 | *0.055* |  |
|  |  | % Time in association | -0.008 | 0.004 | -1.938 | *0.053* |  |
|  |  | **Controls** |  |  |  |  |  |
|  |  | Fruit availability | 0.000 | 0.001 | 0.230 | *0.710* |  |
|  |  | Total waypoints per path | 0.002 | 0.000 | 6.870 | *<0.001* |  |
|  |  | Rainfall | 0.000 | 0.000 | 0.670 | *0.560* |  |
|  |  | **Random Effects** | **Variance** | **SD** |  |  |  |
|  |  | Focal ID (Intercept) | 0.000 | 0.014 |  |  |  |
|  |  | Residual | 0.003 | 0.054 |  |  |  |
| Model_1_No.H | **Number of hotspots visited per day** | **Fixed effects** |  |  |  |  |  |
| *n = 1235 observations;* |  | (Intercept) | 1.033 | 0.140 | 7.379 | <0.001 | 0.013 / 0.027 |
| *87 individuals* |  | Class [Independent Immature] | -0.165 | 0.047 | -3.473 | <0.001 |  |
|  |  | Class [Mother] | -0.137 | 0.048 | -2.871 | 0.004 |  |
|  |  | Class [Unflanged Male] | -0.179 | 0.060 | -3.007 | 0.003 |  |
|  |  | % Time in association | -0.129 | 0.025 | -5.078 | <0.001 |  |
|  |  | **Controls** |  |  |  |  |  |
|  |  | Fruit availability | 0.005 | 0.004 | 1.285 | 0.199 |  |
|  |  | Active period duration | 0.056 | 0.012 | 4.662 | <0.001 |  |
|  |  | Rainfall | -0.001 | 0.001 | -0.942 | 0.346 |  |
|  |  | **Random effects** | **Variance** | **SD** |  |  |  |
|  |  | Focal ID (Intercept) | 0.009 | 0.096 |  |  |  |
|  |  | Residual | NA | NA |  |  |  |
| Model_1_Time | **Log(Time in a hotspot)** | **Fixed effects** |  |  |  |  |  |
| *n = 6571 observations;* |  | (Intercept) | 3.886 | 0.030 | 131.069 | <0.001 | **0.009 / 0.053** |
| *110 individuals;* |  | Class [Independent Immature] | -0.076 | 0.021 | -3.655 | <0.001 |  |
| *2154 follows* |  | Class [Mother] | -0.064 | 0.020 | -3.155 | 0.003 |  |
|  |  | Class [Unflanged Male] | -0.034 | 0.024 | -1.417 | 0.159 |  |
|  |  | % Time in association at hotspot | -0.041 | 0.011 | -3.649 | <0.001 |  |
|  |  | **Controls** |  |  |  |  |  |
|  |  | Fruit availability | 0.003 | 0.002 | 1.772 | 0.077 |  |
|  |  | Active period duration | 0.002 | 0.002 | 1.250 | 0.212 |  |
|  |  | Rainfall | -0.000 | 0.000 | -0.457 | 0.647 |  |
|  |  | **Random effects** | **Variance** | **SD** |  |  |  |
|  |  | Focal ID (Intercept) | 0.006 | 0.078 |  |  |  |
|  |  | Follow ID (Intercept) | 0.001 | 0.036 |  |  |  |
|  |  | Residual | 0.160 | 0.400 |  |  |  |
| **Model2_DPL_UF** | **Daily path length**  *(Unflanged Male)* | **Fixed effects** |  |  |  |  |  |
| *n = 62 observations;* |  | (Intercept) | 1060.112 | 220.375 | 4.811 | *< 0.001* | 0.118 / NA |
| *20 individuals* |  | % Time in association | 291.823 | 131.147 | 2.225 | *0.030* |  |
|  |  | **Controls** |  |  |  |  |  |
|  |  | Fruit availability | 12.879 | 20.835 | 0.666 | *0.508* |  |
|  |  | Rainfall | 15.279 | 9.733 | 1.570 | *0.122* |  |
|  |  | **Random effects** | **Variance** | **SD** |  |  |  |
|  |  | Focal ID (Intercept) | 0.000 | 0.000 |  |  |  |
|  |  | Residual | 254680.000 | 504.700 |  |  |  |
| **Model2_DPL_FL**  *n = 201 observations;*  *17 individuals* | **Daily path length**  *(Flanged Male)* | **Fixed effects** |  |  |  |  |  |
|  |  | (Intercept) | 865.013 | 123.148 | 7.024 | *< 0.001* | **0.040 / 0.076** |
|  |  | % Time in association | 90.643 | 80.540 | 1.125 | *0.262* |  |
|  |  | **Controls** |  |  |  |  |  |
|  |  | Fruit availability | 27.511 | 11.074 | 2.484 | *0.014* |  |
|  |  | Rainfall | -4.369 | 6.095 | -0.717 | *0.474* |  |
|  |  | **Random effects** | **Variance** | **SD** |  |  |  |
|  |  | Focal ID (Intercept) | 9440.000 | 97.160 |  |  |  |
|  |  | Residual | 243866.000 | 493.830 |  |  |  |
| **Model2_DPL_M** | **Daily path length**  *(Mother)* | **Fixed effects** |  |  |  |  |  |
| *n = 500 observations;* |  | (Intercept) | 1093.310 | 74.605 | 14.655 | *< 0.001* | **0.002 / 0.011** |
| *16 individuals* |  | % Time in association | 33.083 | 42.350 | 0.781 | *0.435* |  |
|  |  | **Controls** |  |  |  |  |  |
|  |  | Fruit availability | -1,271 | 6.695 | -0.190 | *0.849* |  |
|  |  | Rainfall | -0.604 | 0.949 | -0.643 | *0.520* |  |
|  |  | **Random effects** | **Variance** | **SD** |  |  |  |
|  |  | Focal ID (Intercept) | 1542.000 | 39.270 |  |  |  |
|  |  | Residual | 165740.000 | 407.110 |  |  |  |
| **Model2_DPL_I** | **Daily path length**  *(Independent Immature)* | **Fixed effects** |  |  |  |  |  |
| *n = 208 observations;* |  | (Intercept) | 1253.880 | 132.185 | 9.486 | *< 0.001* | **0.003 / 0.259** |
| *21 individuals* |  | % Time in association | 49.014 | 65.299 | 0.751 | *0.454* |  |
|  |  | **Controls** |  |  |  |  |  |
|  |  | Fruit availability | -1.876 | 12.401 | -0.151 | *0.880* |  |
|  |  | Rainfall | -0.974 | 3.784 | -0.257 | *0.797* |  |
|  |  | **Random effects** | **Variance** | **SD** |  |  |  |
|  |  | Focal ID (Intercept) | 63156.000 | 251.300 |  |  |  |
|  |  | Residual | 182278.000 | 426.900 |  |  |  |
| **Model2_Sin_UF** | **Daily Sinuosity**  *(Unflanged Male)* | **Fixed effects** |  |  |  |  |  |
| *n = 64 observations;* |  | (Intercept) | 0.124 | 0.067 | 1.869 | *0.067* | **0.205 / 0.281** |
| *20 individuals* |  | % Time in association | -0.007 | 0.014 | -0.462 | *0.646* |  |
|  |  | **Controls** |  |  |  |  |  |
|  |  | Fruit availability | -0.003 | 0.002 | -1.254 | *0.215* |  |
|  |  | Total waypoints per path | 0.003 | 0.001 | 2.782 | *0.007* |  |
|  |  | Rainfall | -0.002 | 0.001 | -2.124 | *0.038* |  |
|  |  | **Random effects** | **Variance** | **SD** |  |  |  |
|  |  | Focal ID (Intercept) | 0.000 | 0.017 |  |  |  |
|  |  | Residual | 0.003 | 0.052 |  |  |  |
| **Model2_Sin_FL** | **Daily Sinuosity**  *(Flanged Male)* | **Fixed effects** |  |  |  |  |  |
| *n = 207 observations;* |  | (Intercept) | 0.150 | 0.041 | 3.671 | *<0.001* | **0.088 / 0.127** |
| *18 individuals* |  | % Time in association | -0.012 | 0.010 | -1.135 | *0.258* |  |
|  |  | **Controls** |  |  |  |  |  |
|  |  | Fruit availability | -0.003 | 0.001 | -2.461 | *0.015* |  |
|  |  | Total waypoints per path | 0.002 | 0.001 | 3.898 | *<0.001* |  |
|  |  | Rainfall | -0.000 | 0.001 | -0.491 | *0.624* |  |
|  |  | **Random effects** | **Variance** | **SD** |  |  |  |
|  |  | Focal ID (Intercept) | 0.000 | 0.013 |  |  |  |
|  |  | Residual | 0.004 | 0.062 |  |  |  |
| **Model2_Sin_M** | **Daily Sinuosity**  *(Mother)* | **Fixed effects** |  |  |  |  |  |
| *n = 508 observations;* |  | (Intercept) | 0.164 | 0.025 | 6.623 | *<0.001* | **0.056 / 0.059** |
| *16 individuals* |  | % Time in association | -0.003 | 0.005 | -0.663 | *0.508* |  |
|  |  | **Controls** |  |  |  |  |  |
|  |  | Fruit availability | 0.001 | 0.001 | 1.757 | *0.080* |  |
|  |  | Total waypoints per path | 0.002 | 0.000 | 5.036 | *<0.001* |  |
|  |  | Rainfall | 0.000 | 0.000 | 1.426 | *0.154* |  |
|  |  | **Random effects** | **Variance** | **SD** |  |  |  |
|  |  | Focal ID (Intercept) | 0.000 | 0.003 |  |  |  |
|  |  | Residual | 0.002 | 0.050 |  |  |  |
| **Model2_Sin_I** | **Daily Sinuosity**  *(Independent Immature)* | **Fixed effects** |  |  |  |  |  |
| *n = 213 observations;* |  | (Intercept) | 0.230 | 0.038 | 6.050 | *<0.001* | **0.027 / 0.164** |
| *22 individuals* |  | % Time in association | -0.012 | 0.008 | -1.523 | *0.129* |  |
|  |  | **Controls** |  |  |  |  |  |
|  |  | Fruit availability | 0.001 | 0.001 | 0.420 | *0.675* |  |
|  |  | Total waypoints per path | 0.001 | 0.001 | 1.348 | *0.179* |  |
|  |  | Rainfall | -0.001 | 0.000 | -1.260 | *0.209* |  |
|  |  | **Random effects** | **Variance** | **SD** |  |  |  |
|  |  | Focal ID (Intercept) | 0.000 | 0.021 |  |  |  |
|  |  | Residual | 0.003 | 0.051 |  |  |  |
| Model2_No.H_UF | **Number of hotspots visited per day**  *(Unflanged Male)* | **Fixed effects** |  |  |  |  |  |
| *n = 89 observations;* |  | (Intercept) | 0.702 | 0.523 | 1.341 | *0.180* | 0.015 / NA |
| *26 individuals* |  | % Time in association | -0.173 | 0.093 | -1.864 | *0.062* |  |
|  |  | **Controls** |  |  |  |  |  |
|  |  | Fruit availability | 0.013 | 0.015 | 0.827 | *0.408* |  |
|  |  | Active period duration | 0.065 | 0.044 | 1.470 | *0.142* |  |
|  |  | Rainfall | -0.007 | 0.007 | -1.038 | *0.299* |  |
|  |  | **Random effects** | **Variance** | **SD** |  |  |  |
|  |  | Focal ID (Intercept) | 0.000 | 0.000 |  |  |  |
|  |  | Residual | NA | NA |  |  |  |
| Model2_No.H_FL | **Number of hotspots visited per day**  *(Flanged Male)* | **Fixed effects** |  |  |  |  |  |
| *n = 250 observations;* |  | (Intercept) | 0.829 | 0.238 | 3.488 | *<0.001* | 0.016 / 0.022 |
| *23 individuals* |  | % Time in association | -0.116 | 0.050 | -2.325 | *0.020* |  |
|  |  | **Controls** |  |  |  |  |  |
|  |  | Fruit availability | 0.012 | 0.007 | 1.867 | *0.062* |  |
|  |  | Active period duration | 0.068 | 0.022 | 3.158 | *0.002* |  |
|  |  | Rainfall | 0.002 | 0.004 | 0.421 | *0.674* |  |
|  |  | **Random effects** | **Variance** | **SD** |  |  |  |
|  |  | Focal ID (Intercept) | 0.004 | 0.061 |  |  |  |
|  |  | Residual | NA | NA |  |  |  |
| Model2_No.H_M | **Number of hotspots visited per day**  *(Mother)* | **Fixed effects** |  |  |  |  |  |
| *n = 615 observations;* |  | (Intercept) | 0.822 | 0.232 | 3.546 | *<0.001* | 0.008 / 0.021 |
| *19 individuals* |  | % Time in association | -0.125 | 0.040 | -3.110 | *0.002* |  |
|  |  | **Controls** |  |  |  |  |  |
|  |  | Fruit availability | -0.000 | 0.006 | -0.028 | *0.977* |  |
|  |  | Active period duration | 0.066 | 0.020 | 3.330 | *<0.001* |  |
|  |  | Rainfall | -0.001 | 0.001 | -0.626 | *0.531* |  |
|  |  | **Random effects** | **Variance** | **SD** |  |  |  |
|  |  | Focal ID (Intercept) | 0.011 | 0.105 |  |  |  |
|  |  | Residual | NA | NA |  |  |  |
| Model2_No.H_I | **Number of hotspots visited per day**  *(Independent immature)* | **Fixed effects** |  |  |  |  |  |
| *n = 281 observations;* |  | (Intercept) | 1.282 | 0.285 | 4.505 | *<0.001* | 0.008 / 0.035 |
| *25 individuals* |  | % Time in association | -0.121 | 0.047 | -2.563 | *0.010* |  |
|  |  | **Controls** |  |  |  |  |  |
|  |  | Fruit availability | 0.005 | 0.009 | 0.558 | *0.577* |  |
|  |  | Active period duration | 0.021 | 0.024 | 0.883 | *0.377* |  |
|  |  | Rainfall | -0.003 | 0.003 | -1.024 | *0.306* |  |
|  |  | **Random effects** | **Variance** | **SD** |  |  |  |
|  |  | Focal ID (Intercept) | 0.016 | 0.127 |  |  |  |
|  |  | Residual | NA | NA |  |  |  |
| **Model2_Time_UF** | **log(Time in a hotspot)** *(Unflanged Male)* | **Fixed effects** |  |  |  |  |  |
| *n = 610 observations;* |  | (Intercept) | 3.907 | 0.075 | 52.093 | <0.001 | NA / 0.009 |
| *39 individuals;* |  | % Time in association at hotspot | -0.060 | 0.034 | -1.779 | 0.076 |  |
| *259 follows* |  | **Controls** |  |  |  |  |  |
|  |  | Fruit availability | -0.004 | 0.006 | -0.717 | 0.474 |  |
|  |  | Active period duration | 0.006 | 0.005 | 1.103 | 0.271 |  |
|  |  | Rainfall | -0.001 | 0.002 | -0.691 | 0.490 |  |
|  |  | **Random effects** | **Variance** | **SD** |  |  |  |
|  |  | Focal ID (Intercept) | 0.000 | 0.000 |  |  |  |
|  |  | Follow ID (Intercept) | 0.001 | 0.035 |  |  |  |
|  |  | Residual | 0.156 | 0.394 |  |  |  |
| **Model2_Time_FL** | **log(Time in a hotspot)** *(Flanged Male)* | **Fixed effects** |  |  |  |  |  |
| *n = 1664 observations;* |  | (Intercept) | 3.829 | 0.055 | 69.340 | *<0.001* | 0.010 / 0.076 |
| *27 individuals;* |  | % Time in association at hotspot | -0.079 | 0.026 | -3.030 | *0.003* |  |
| *527 follows* |  | **Controls** |  |  |  |  |  |
|  |  | Fruit availability | 0.005 | 0.003 | 1.605 | *0.110* |  |
|  |  | Active period duration | 0.007 | 0.004 | 1.725 | *0.085* |  |
|  |  | Rainfall | 0.001 | 0.001 | 0.762 | *0.447* |  |
|  |  | **Random effects** | **Variance** | **SD** |  |  |  |
|  |  | Focal ID (Intercept) | 0.004 | 0.064 |  |  |  |
|  |  | Follow ID (Intercept) | 0.008 | 0.087 |  |  |  |
|  |  | Residual | 0.177 | 0.421 |  |  |  |
| **Model2_Time_M** | **log(Time in a hotspot)** *(Mother)* | **Fixed effects** |  |  |  |  |  |
| *n = 2932 observations;* |  | (Intercept) | 3.816 | 0.045 | 84.839 | *<0.001* | 0.001 / 0.047 |
| *22 individuals;* |  | % Time in association at hotspot | -0.007 | 0.016 | -0.440 | *0.660* |  |
| *899 follows* |  | **Controls** |  |  |  |  |  |
|  |  | Fruit availability | 0.003 | 0.003 | 1.210 | *0.228* |  |
|  |  | Active period duration | 0.001 | 0.003 | 0.290 | *0.770* |  |
|  |  | Rainfall | 0.000 | 0.000 | -1.000 | *0.321* |  |
|  |  | **Random effects** | **Variance** | **SD** |  |  |  |
|  |  | Focal ID (Intercept) | 0.001 | 0.023 |  |  |  |
|  |  | Follow ID (Intercept) | 0.007 | 0.081 |  |  |  |
|  |  | Residual | 0.157 | 0.396 |  |  |  |
| **Model2_Time_I** | **log(Time in a hotspot)**  *(Independent Immature)* | **Fixed effects** |  |  |  |  |  |
| *n = 1365 observations;* |  | (Intercept) | 3.874 | 0.058 | 67.222 | *<0.001* | 0.008 / 0.049 |
| *30 individuals;* |  | % Time in association at hotspot | -0.065 | 0.023 | -2.832 | *0.005* |  |
| *469 follows* |  | **Controls** |  |  |  |  |  |
|  |  | Fruit availability | 0.002 | 0.004 | 0.470 | *0.621* |  |
|  |  | Active period duration | -0.002 | 0.004 | -0.460 | *0.648* |  |
|  |  | Rainfall | 0.002 | 0.002 | 1.050 | *0.297* |  |
|  |  | **Random effects** | **Variance** | **SD** |  |  |  |
|  |  | Focal ID (Intercept) | 0.001 | 0.026 |  |  |  |
|  |  | Follow ID (Intercept) | 0.005 | 0.071 |  |  |  |
|  |  | Residual | 0.144 | 0.380 |  |  |  |
| **Model3_DPL_UF** | **Daily Path Length**  *(Unflanged Male)* | **Fixed effects** |  |  |  |  |  |
| *n = 28 observations;* |  | (Intercept) | 1425.462 | 405.335 | 3.517 | *0.002* | **0.184 / 0.218** |
| *13 individuals* |  | Presence Unflanged | -76.973 | 186.553 | -0.413 | *0.685* |  |
|  |  | Presence Flanged | 70.761 | 251.681 | 0.281 | *0.782* |  |
|  |  | Presence Mother | 308.720 | 233.137 | 1.324 | *0.201* |  |
|  |  | Presence Immature | -116.571 | 262.229 | -0.446 | *0.660* |  |
|  |  | **Controls** |  |  |  |  |  |
|  |  | Fruit availability | -7.372 | 27.751 | -0.266 | *0.793* |  |
|  |  | Rainfall | 11.472 | 16.134 | 0.711 | *0.486* |  |
|  |  | **Random effects** | **Variance** | **SD** |  |  |  |
|  |  | Focal ID (Intercept) | 7494.000 | 86.57 |  |  |  |
|  |  | Residual | 177300.000 | 421.07 |  |  |  |
| **Model3_DPL_FL** | **Daily Path Length** *(Flanged Male)* | **Fixed effects** |  |  |  |  |  |
| *n = 62 observations;* |  | (Intercept) | 969.040 | 190.248 | 5.094 | *<0.001* | **0.055 / NA** |
| *14 individuals* |  | Presence Unflanged | 117.439 | 219.719 | 0.534 | *0.595* |  |
|  |  | Presence Mother | 0.267 | 122.163 | 0.002 | *0.998* |  |
|  |  | Presence Immature | 23.230 | 132.755 | 0.175 | *0.862* |  |
|  |  | **Controls** |  |  |  |  |  |
|  |  | Fruit availability | 25.606 | 16.426 | 1.559 | *0.125* |  |
|  |  | Rainfall | -10.347 | 12.618 | -0.820 | *0.416* |  |
|  |  | **Random effects** | **Variance** | **SD** |  |  |  |
|  |  | Focal ID (Intercept) | 0.000 | 0.000 |  |  |  |
|  |  | Residual | 158359.000 | 397.900 |  |  |  |
| **Model3_DPL_M** | **Daily Path Length** *(Mother)* | **Fixed effects** |  |  |  |  |  |
| *n = 487 observations;* |  | (Intercept) | 1067.415 | 70.168 | 15.212 | *<0.001* | **0.030 / NA** |
| *16 individuals* |  | Presence Unflanged | 49.090 | 39.470 | 1.244 | *0.214* |  |
|  |  | Presence Flanged | 150.916 | 54.271 | 2.781 | *0.006* |  |
|  |  | Presence Mother | 68.382 | 47.844 | 1.429 | *0.154* |  |
|  |  | Presence Immature | -36.500 | 46.543 | -0.784 | *0.443* |  |
|  |  | **Controls** |  |  |  |  |  |
|  |  | Fruit availability | -1.515 | 6.624 | -0.229 | *0.819* |  |
|  |  | Rainfall | -0.653 | 0.928 | -0.703 | *0.482* |  |
|  |  | **Random effects** | **Variance** | **SD** |  |  |  |
|  |  | Focal ID (Intercept) | 0.000 | 0.000 |  |  |  |
|  |  | Residual | 161135.000 | 401.400 |  |  |  |
| **Model3_DPL_I** | **Daily Path Length** *(Independent Immature)* | **Fixed effects** |  |  |  |  |  |
| *n = 114 observations;* |  | (Intercept) | 1438.208 | 175.308 | 8.204 | *<0.001* | **0.030 / 0.209** |
| *20 individuals* |  | Presence Unflanged | 6.002 | 84.696 | 0.071 | *0.944* |  |
|  |  | Presence Flanged | 137.547 | 116.906 | 1.177 | *0.242* |  |
|  |  | Presence Mother | 16.063 | 90.255 | 0.178 | *0.859* |  |
|  |  | Presence Immature | -4.041 | 104.576 | -0.039 | *0.969* |  |
|  |  | **Controls** |  |  |  |  |  |
|  |  | Fruit availability | -21.758 | 15.226 | -1.429 | *0.156* |  |
|  |  | Rainfall | -4.198 | 5.111 | -0.821 | *0.413* |  |
|  |  | **Random effects** | **Variance** | **SD** |  |  |  |
|  |  | Focal ID (Intercept) | 36731.000 | 191.700 |  |  |  |
|  |  | Residual | 161942.000 | 402.200 |  |  |  |
| **Model3_Sin_UF** | **Daily Sinuosity**  *(Unflanged Male)* | **Fixed effects** |  |  |  |  |  |
| *n = 28 observations;* |  | (Intercept) | 0.001 | 0.116 | -0.044 | *0.965* | **0.427 / 0.642** |
| *13 individuals* |  | Presence Unflanged | 0.007 | 0.018 | 0.375 | *0.712* |  |
|  |  | Presence Flanged | 0.243 | 0.024 | 1.019 | *0.322* |  |
|  |  | Presence Mother | -0.014 | 0.022 | -0.623 | *0.541* |  |
|  |  | Presence Immature | 0.061 | 0.026 | 2.361 | *0.025* |  |
|  |  | **Controls** |  |  |  |  |  |
|  |  | Fruit availability | -0.002 | 0.003 | -0.550 | *0.589* |  |
|  |  | Total waypoints per path | 0.004 | 0.001 | 2.861 | *0.008* |  |
|  |  | Rainfall | -0.003 | 0.002 | -2.093 | *0.046* |  |
|  |  | **Random effects** | **Variance** | **SD** |  |  |  |
|  |  | Focal ID (Intercept) | 0.001 | 0.027 |  |  |  |
|  |  | Residual | 0.001 | 0.035 |  |  |  |
| **Model3_Sin_FL** | **Daily Sinuosity**  *(Flanged Male)* | **Fixed effects** |  |  |  |  |  |
| *n = 65 observations;* |  | (Intercept) | 0.127 | 0.054 | 2.337 | *0.023* | **0.195 / NA** |
| *14 individuals* |  | Presence Unflanged | -0.039 | 0.027 | -1.431 | *0.158* |  |
|  |  | Presence Mother | 0.004 | 0.015 | 0.250 | *0.804* |  |
|  |  | Presence Immature | 0.010 | 0.017 | 0.571 | *0.571* |  |
|  |  | **Controls** |  |  |  |  |  |
|  |  | Fruit availability | -0.004 | 0.002 | -1.869 | *0.067* |  |
|  |  | Total waypoints per path | 0.003 | 0.001 | 2.296 | *0.005* |  |
|  |  | Rainfall | 0.003 | 0.002 | 1.601 | *0.115* |  |
|  |  | **Random effects** | **Variance** | **SD** |  |  |  |
|  |  | Focal ID (Intercept) | 0.000 | 0.000 |  |  |  |
|  |  | Residual | 0.002 | 0.050 |  |  |  |
| **Model3_Sin_M** | **Daily Sinuosity**  *(Mother)* | **Fixed effects** |  |  |  |  |  |
| *n = 495 observations;* |  | (Intercept) | 0.139 | 0.025 | 5.485 | *<0.001* | **0.077 / 0.084** |
| *16 individuals* |  | Presence Unflanged | -0.005 | 0.005 | -0.994 | *0.321* |  |
|  |  | Presence Flanged | -0.013 | 0.007 | -2.002 | *0.046* |  |
|  |  | Presence Mother | -0.005 | 0.006 | -0.763 | *0.466* |  |
|  |  | Presence Immature | 0.003 | 0.006 | 0.600 | *0.549* |  |
|  |  | **Controls** |  |  |  |  |  |
|  |  | Fruit availability | 0.002 | 0.001 | 2.080 | *0.038* |  |
|  |  | Total waypoints per path | 0.002 | 0.000 | 5.817 | *<0.001* |  |
|  |  | Rainfall | 0.000 | 0.000 | 1.642 | *0.101* |  |
|  |  | **Random effects** | **Variance** | **SD** |  |  |  |
|  |  | Focal ID (Intercept) | 0.000 | 0.004 |  |  |  |
|  |  | Residual | 0.002 | 0.049 |  |  |  |
| **Model3_Sin_I** | **Daily Sinuosity**  *(Independent Immature)* | **Fixed effects** |  |  |  |  |  |
| *n = 119 observations;* |  | (Intercept) | 0.271 | 0.040 | 6.700 | *<0.001* | **0.059 / 0.124** |
| *21 individuals* |  | Presence Unflanged | -0.006 | 0.008 | -0.771 | *0.442* |  |
|  |  | Presence Flanged | -0.022 | 0.011 | -1.991 | *0.049* |  |
|  |  | Presence Mother | 0.010 | 0.009 | 1.111 | *0.269* |  |
|  |  | Presence Immature | 0.007 | 0.010 | 0.641 | *0.523* |  |
|  |  | **Controls** |  |  |  |  |  |
|  |  | Fruit availability | 0.001 | 0.001 | 0.702 | *0.484* |  |
|  |  | Total waypoints per path | -0.000 | 0.001 | -0.299 | *0.766* |  |
|  |  | Rainfall | 0.000 | 0.001 | 0.426 | *0.671* |  |
|  |  | **Random effects** | **Variance** | **SD** |  |  |  |
|  |  | Focal ID (Intercept) | 0.000 | 0.011 |  |  |  |
|  |  | Residual | 0.002 | 0.042 |  |  |  |
| Model3_No.H_UF | **Number of hotspots visited per day**  *(Unflanged male)* | **Fixed effects** |  |  |  |  |  |
| *n = 44 observations;* |  | (Intercept) | 1.607 | 0.903 | 1.780 | *0.075* | 0.026 / 0.027 |
| *18 individuals* |  | Presence Unflanged | -0.064 | 0.149 | -0.432 | *0.666* |  |
|  |  | Presence Flanged | 0.049 | 0.170 | 0.289 | *0.773* |  |
|  |  | Presence Mother | -0.204 | 0.156 | -1.311 | *0.190* |  |
|  |  | Presence Immature | -0.178 | 0.168 | -1.059 | *0.290* |  |
|  |  | **Controls** |  |  |  |  |  |
|  |  | Fruit availability | 0.009 | 0.025 | 0.355 | *0.723* |  |
|  |  | Active period duration | -0.007 | 0.065 | -0.109 | *0.913* |  |
|  |  | Rainfall | 0.009 | 0.014 | 0.630 | *0.529* |  |
|  |  | **Random effects** | **Variance** | **SD** |  |  |  |
|  |  | Focal ID (Intercept) | 0.000 | 0.000 |  |  |  |
| Model3_No.H_FL | **Number of hotspots visited per day**  *(Flanged male)* | **Fixed effects** |  |  |  |  |  |
| *n = 90 observations;* |  | (Intercept) | 0.749 | 0.389 | 1.925 | *0.054* | 0.026 / 0.027 |
| *19 individuals* |  | Presence Unflanged | -0.196 | 0.137 | -1.433 | *0.152* |  |
|  |  | Presence Flanged | -0.073 | 0.178 | -0.409 | *0.683* |  |
|  |  | Presence Mother | 0.088 | 0.078 | 1.124 | *0.261* |  |
|  |  | Presence Immature | 0.076 | 0.092 | 0.828 | *0.408* |  |
|  |  | **Controls** |  |  |  |  |  |
|  |  | Fruit availability | 0.010 | 0.012 | 0.867 | *0.386* |  |
|  |  | Active period duration | 0.062 | 0.035 | 1.762 | *0.078* |  |
|  |  | Rainfall | 0.008 | 0.008 | 0.990 | *0.322* |  |
|  |  | **Random effects** | **Variance** | **SD** |  |  |  |
|  |  | Focal ID (Intercept) | 0.001 | 0.023 |  |  |  |
| Model3_No.H_M | **Number of hotspots visited per day**  *(Mother)* | **Fixed effects** |  |  |  |  |  |
| *n = 600 observations;* |  | (Intercept) | 0.646 | 0.238 | 2.717 | *0.007* | 0.011 / 0.022 |
| *19 individuals* |  | Presence Unflanged | -0.116 | 0.038 | -3.039 | *0.002* |  |
|  |  | Presence Flanged | -0.102 | 0.051 | -2.015 | *0.044* |  |
|  |  | Presence Mother | -0.061 | 0.046 | -1.349 | *0.177* |  |
|  |  | Presence Immature | 0.018 | 0.042 | 0.433 | *0.665* |  |
|  |  | **Controls** |  |  |  |  |  |
|  |  | Fruit availability | 0.003 | 0.006 | 0.501 | *0.616* |  |
|  |  | Active period duration | 0.080 | 0.020 | 3.900 | *<0.001* |  |
|  |  | Rainfall | -0.000 | 0.001 | -0.461 | *0.645* |  |
|  |  | **Random effects** | **Variance** | **SD** |  |  |  |
|  |  | Focal ID (Intercept) | 0.009 | 0.092 |  |  |  |
| Model3_No.H_I | **Number of hotspots visited per day**  *(Independent immature)* | **Fixed effects** |  |  |  |  |  |
| *n = 163 observations;* |  | (Intercept) | 0.882 | 0.418 | 2.111 | *0.035* | 0.017 / 0.076 |
| *24 individuals* |  | Presence Unflanged | -0.104 | 0.071 | -1.453 | *0.146* |  |
|  |  | Presence Flanged | 0.058 | 0.092 | 0.632 | *0.527* |  |
|  |  | Presence Mother | -0.130 | 0.075 | -1.727 | *0.084* |  |
|  |  | Presence Immature | -0.125 | 0.083 | -1.505 | *0.132* |  |
|  |  | **Controls** |  |  |  |  |  |
|  |  | Fruit availability | 0.004 | 0.012 | 0.372 | *0.710* |  |
|  |  | Active period duration | 0.058 | 0.034 | 1.680 | *0.093* |  |
|  |  | Rainfall | -0.005 | 0.005 | -0.947 | *0.344* |  |
|  |  | **Random effects** | **Variance** | **SD** |  |  |  |
|  |  | Focal ID (Intercept) | 0.039 | 0.198 |  |  |  |
| Model3_Time_UF | **log(Time in a hotspot)** *(Unflanged male)* | **Fixed effects** |  |  |  |  |  |
| *n = 610 observations;* |  | (Intercept) | 3.898 | 0.077 | 50.636 | *<0.001* | 0.008 / 0.018 |
| *39 individuals;* |  | Presence Unflanged | 0.030 | 0.068 | 0.445 | *0.657* |  |
| *259 follows* |  | Presence Flanged | -0.030 | 0.097 | -0.310 | *0.757* |  |
|  |  | Presence Mother | -0.067 | 0.049 | -1.366 | *0.173* |  |
|  |  | Presence Immature | 0.002 | 0.052 | 0.041 | *0.967* |  |
|  |  | **Controls** |  |  |  |  |  |
|  |  | Fruit availability | -0.004 | 0.006 | -0.773 | *0.440* |  |
|  |  | Active period duration | 0.004 | 0.005 | 0.964 | *0.336* |  |
|  |  | Rainfall | -0.001 | 0.002 | -0.551 | *0.582* |  |
|  |  | **Random effects** | **Variance** | **SD** |  |  |  |
|  |  | Focal ID (Intercept) | 0.000 | 0.000 |  |  |  |
|  |  | Follow ID (Intercept) | 0.002 | 0.040 |  |  |  |
|  |  | Residual | 0.156 | 0.395 |  |  |  |
| Model3_Time_FL | **log(Time in a hotspot)** *(Flanged male)* | **Fixed effects** |  |  |  |  |  |
| *n = 1664 observations;* |  | (Intercept) | 3.813 | 0.056 | 67.116 | *<0.001* | 0.004 / 0.078 |
| *27 individuals;* |  | Presence Unflanged | 0.009 | 0.154 | -0.062 | *0.951* |  |
| *527 follows* |  | Presence Flanged | -0.049 | 0.251 | -0.197 | *0.884* |  |
|  |  | Presence Mother | 0.038 | 0.046 | 0.714 | *0.476* |  |
|  |  | Presence Immature | 0.034 | 0.060 | 0.559 | *0.576* |  |
|  |  | **Controls** |  |  |  |  |  |
|  |  | Fruit availability | 0.006 | 0.003 | 1.817 | *0.070* |  |
|  |  | Active period duration | 0.005 | 0.004 | 1.249 | *0.212* |  |
|  |  | Rainfall | -0.001 | 0.001 | 0.558 | *0.577* |  |
|  |  | **Random effects** | **Variance** | **SD** |  |  |  |
|  |  | Focal ID (Intercept) | 0.010 | 0.098 |  |  |  |
|  |  | Follow ID (Intercept) | 0.005 | 0.068 |  |  |  |
|  |  | Residual | 0.177 | 0.421 |  |  |  |
| Model3_Time_M | **log(Time in a hotspot)** *(Mother)* | **Fixed effects** |  |  |  |  |  |
| *n = 2932 observations;* |  | (Intercept) | 3.798 | 0.045 | 84.767 | *<0.001* | 0.007 / 0.049 |
| *22 individuals;* |  | Presence Unflanged | 0.019 | 0.021 | 0.881 | *0.379* |  |
| *899 follows;* |  | Presence Flanged | 0.123 | 0.034 | 3.592 | *<0.001* |  |
|  |  | Presence Mother | -0.061 | 0.028 | -2.120 | *0.034* |  |
|  |  | Presence Immature | -0.015 | 0.023 | -0.648 | *0.517* |  |
|  |  | **Controls** |  |  |  |  |  |
|  |  | Fruit availability | 0.004 | 0.003 | 1.431 | *0.153* |  |
|  |  | Active period duration | 0.001 | 0.003 | 0.413 | *0.680* |  |
|  |  | Rainfall | -0.000 | 0.000 | -1.161 | *0.246* |  |
|  |  | **Random effects** | **Variance** | **SD** |  |  |  |
|  |  | Focal ID (Intercept) | 0.006 | 0.077 |  |  |  |
|  |  | Follow ID (Intercept) | 0.001 | 0.030 |  |  |  |
|  |  | Residual | 0.157 | 0.396 |  |  |  |
| Model3_Time_I | **log(Time in a hotspot)** (*Independent immature*) | **Fixed effects** |  |  |  |  |  |
| *n = 1365 observations;* |  | (Intercept) | 3.851 | 0.044 | 65.400 | *<0.001* | 0.008 / 0.054 |
| *30 individuals;* |  | Presence Unflanged | -0.004 | 0.055 | -0.111 | *0.912* |  |
| *469 follows;* |  | Presence Flanged | 0.062 | 0.049 | 1.293 | *0.197* |  |
|  |  | Presence Mother | -0.076 | 0.042 | -2.337 | *0.020* |  |
|  |  | Presence Immature | -0.081 | 0.062 | -0.018 | *0.985* |  |
|  |  | **Controls** |  |  |  |  |  |
|  |  | Fruit availability | 0.001 | 0.026 | 0.315 | *0.753* |  |
|  |  | Active period duration | -0.002 | 0.072 | -0.460 | *0.646* |  |
|  |  | Rainfall | 0.002 | 0.045 | 0.964 | *0.336* |  |
|  |  | **Random effects** | **Variance** | **SD** |  |  |  |
|  |  | Focal ID (Intercept) | 0.000 | 0.000 |  |  |  |
|  |  | Follow ID (Intercept) | 0.008 | 0.089 |  |  |  |
|  |  | Residual | 0.107 | 0.328 |  |  |  |
