# Supplementary material for: The immediate social environment dynamically shapes movement and foraging in wild Sumatran orangutans (Pongo abelii)
Source: Mov Ecol. 2026 Oct 21;14:74. doi: 10.1186/s40462-026-00713-2 (PMC13689139; doi:10.1186/s40462-026-00713-2)
Supplement: Supplementary file 4 [file 40462_2026_713_MOESM4_ESM.docx]

***Supplementary table 4. Summary statistics.*** *Descriptive statistics for response and predictor variables across the study population and by age-sex class. Values are observed means ± SD, with the sample size (n) in parentheses.*

| Variable | Population | Independent Immature | Mother | Unflanged Male | Flanged Male |
| --- | --- | --- | --- | --- | --- |
| % Time in association | **41.99** ± 46.06  *(n = 981)* | **49.44** ± 49.58  *(n = 202)* | **44.72** ± 43.59  *(n = 508)* | **45.31** ± 50.17  *(n = 64)* | **27.02** ± 44.13  *(n = 207)* |
| Active period duration (h) | **11.28** ± 1.00  *(n = 1223)* | **11.35** ± 1.03  *(n = 269)* | **11.36** ± 0.92  *(n = 615)* | **11.37** ± 1.04  *(n = 89)* | **10.98** ± 1.08  *(n = 250)* |
| Daily path length (m/day) | **1154.90** ± 466.16  *(n = 960)* | **1236.32** ± 495.18  *(n = 197)* | **1092.80** ± 409.45  *(n = 500)* | **1385.29** ± 541.32  *(n = 62)* | **1158.52** ± 511.26  *(n = 201)* |
| Daily path sinuosity | **0.28** ± 0.06  *(n = 981)* | **0.28** ± 0.06  *(n = 202)* | **0.29** ± 0.05  *(n = 508)* | **0.25** ± 0.06  *(n = 64)* | **0.27** ± 0.07  *(n = 207)* |
| Hotspots visited/day | **4.77** ± 1.90  *(n = 1223)* | **4.54** ± 1.76  *(n = 269)* | **4.72** ± 1.96  *(n = 615)* | **4.34** ± 1.72  *(n = 89)* | **5.30** ± 1.83  *(n = 250)* |
| Time in hotspot (min) | **53.94** ± 23.21  *(n = 2136)* | **51.18** ± 19.02  *(n = 451)* | **53.00** ± 23.41  *(n = 899)* | **52.94** ± 22.61  *(n = 259)* | **58.39** ± 25.72  *(n = 527)* |
