# Supplementary material for: The immediate social environment dynamically shapes movement and foraging in wild Sumatran orangutans (Pongo abelii)
Source: Mov Ecol. 2026 Oct 21;14:74. doi: 10.1186/s40462-026-00713-2 (PMC13689139; doi:10.1186/s40462-026-00713-2)
Supplement: Supplementary file 5 [file 40462_2026_713_MOESM5_ESM.docx]

***Supplementary table 5. Effects of active period duration on orangutan daily path length***

| **Model1_DPL:  lmer(DPL ~ Class + TimeInAssociation + FruitAvailability + Rainfall + (1 \| focalID)** | | | |
| --- | --- | --- | --- |
| **Predictor** | **Estimate ± SE** | **t** | **p** |
| (Intercept) | 1033.60 ± 74.96 | 13.79 | <0.001 |
| Class: Independent Immature | 78.07 ± 73.16 | 1.07 | 0.286 |
| Class: Mother | -25.90 ± 74.69 | -0.35 | 0.728 |
| Class: Unflanged Male | 200.29 ± 87.66 | 2.29 | 0.022 |
| Time in Association | 68.67 ± 32.07 | 2.14 | 0.032 |
| Fruit Availability | 10.08 ± 5.11 | 1.97 | 0.049 |
| Rainfall | -0.55 ± 0.96 | -0.58 | 0.556 |
| *Model fit: AIC = 14643.4, BIC = 14687.3, logLik = -7312.7* | | |  |
| **Model1b_DPL:  lmer(DPL ~ Class + TimeInAssociation + FruitAvailability + ActivePeriodDuration + Rainfall + (1 \| focalID)** | | | |
| **Predictor** | **Estimate ± SE** | **t** | **p** |
| (Intercept) | -24.38 ± 182.47 | -0.13 | 0.894 |
| Class: Independent Immature | 36.30 ± 66.04 | 0.55 | 0.583 |
| Class: Mother | -82.80 ± 66.85 | -1.24 | 0.215 |
| Class: Unflanged Male | 179.66 ± 81.34 | 2.21 | 0.027 |
| Time in Association | 33.01 ± 31.93 | 1.03 | 0.301 |
| Fruit Availability | 9.01 ± 5.00 | 1.80 | 0.071 |
| Active Period Duration | 98.90 ± 15.71 | 6.29 | <0.001 |
| Rainfall | 0.30 ± 0.96 | 0.31 | 0.755 |
| *Model fit: AIC = 14607.1, BIC = 14655.9, logLik = -7293.6* | | |  |

To account for the fact that CTSD-based movement estimates are already scaled by observation time, our primary model (Model1_DPL) excluded follow duration and included only social and ecological predictors. This specification aligns with the CTSD approach, in which path length is inherently expressed as movement per unit time, making additional inclusion of follow duration potentially redundant.

To assess whether association effects were nevertheless mediated through remaining variation in active period duration, we also fitted an alternative model (Model1b_DPL) including active period duration. Model1b_DPL provided a substantially improved fit to the data compared to Model_DPL (likelihood ratio test: χ² = 38.27, df = 1, p < 0.001), with a lower AIC (14607.1 vs. 14643.4). In the extended model, active period duration was a strong positive predictor of distance travelled (β = 98.90 ± 15.71, t = 6.2), whereas the effect of Time in Association was no longer statistically significant. Together, these results indicate that individuals in association do not travel farther per unit time; instead, association is linked to longer active (follow) periods, which in turn drive increases in total daily path length.
