# Supplementary material for: The immediate social environment dynamically shapes movement and foraging in wild Sumatran orangutans (Pongo abelii)
Source: Mov Ecol. 2026 Oct 21;14:74. doi: 10.1186/s40462-026-00713-2 (PMC13689139; doi:10.1186/s40462-026-00713-2)
Supplement: Supplementary file 6 [file 40462_2026_713_MOESM6_ESM.docx]

***Supplementary table 6. Effects of active period duration and GPS sampling resolution on path sinuosity.***

| ***Predictor*** | ***Estimate*** | ***SE*** | ***t value*** |
| --- | --- | --- | --- |
| *Intercept* | *0.2662* | *0.0208* | *12.78* |
| *Observation duration (Active Period Duration)* | *0.000804* | *0.00183* | *0.44* |
| *Residual waypoint number (Sampling resolution)* | *0.003864* | *0.000388* | *9.97* |
| ***Random effects*** |  |  |  |
| *Focal ID (intercept) variance = 0.000405 (SD = 0.0201)*  *Residual variance = 0.002764 (SD = 0.0526)* |  |  |  |

*Linear mixed model testing the effects of active period duration and residual waypoint number on path sinuosity. Residual waypoint number represents variation in sampling resolution independent of active period duration. n = 992 observations, 72 individuals.*

Including total waypoint number significantly improved model fit for path sinuosity (likelihood ratio test comparing Model1_Sin with and without number of waypoints (sampling resolution): χ² = 42.86, df = 1, p < 0.001). However, waypoint number was strongly correlated with observation duration due to fixed sampling intervals (Pearson’s r = 0.77, p < 0.001), indicating that its effect largely reflects longer follows rather than increased sampling resolution.

To disentangle these effects, we regressed total waypoint number against active period duration and used the residuals as a measure of sampling resolution independent of active period duration. In a combined model including both predictors, residual waypoint number had a strong positive effect on path, whereas active period duration had no detectable effect (*Supplementary table 6)*. These results indicate that path sinuosity is driven by GPS sampling resolution rather than active period duration, with higher-resolution tracks yielding greater estimated sinuosity.
