# Supplementary material for: The immediate social environment dynamically shapes movement and foraging in wild Sumatran orangutans (Pongo abelii)
Source: Mov Ecol. 2026 Oct 21;14:74. doi: 10.1186/s40462-026-00713-2 (PMC13689139; doi:10.1186/s40462-026-00713-2)
Supplement: Supplementary file 7 [file 40462_2026_713_MOESM7_ESM.docx]

***Supplementary figure 1.*** ***Suaq Balimbing study site map.*** *Map of study site with black lines denoting transects (i.e., cleared, narrow foot paths) to assist navigation in the forest, orange lines denoting the transects used for the phenology surveys, and blue line denoting the river. Transects are displayed in UTM coordinates (Easting and Northing; WGS84 UTM Zone 47N, EPSG:32647). The scale bar indicates distance in meters, and the north arrow indicates geographic orientation.*
