# Supplementary material for: The immediate social environment dynamically shapes movement and foraging in wild Sumatran orangutans (Pongo abelii)
Source: Mov Ecol. 2026 Oct 21;14:74. doi: 10.1186/s40462-026-00713-2 (PMC13689139; doi:10.1186/s40462-026-00713-2)
Supplement: Supplementary file 8 [file 40462_2026_713_MOESM8_ESM.docx]

***Supplementary method 1. Sensitivity analysis using a socially weighted hotspot definition.***

We considered whether hotspot identification should account for potential effects of social foraging on resource depletion. In addition to our original definition of a hotspot as a location where a focal individual remained for ≥30 minutes, we generated a socially weighted hotspot metric that incorporated both the feeding duration and the mean number of individuals present. This approach was intended to account for the possibility that patches exploited by multiple individuals may be depleted more rapidly than patches used alone. Weighted hotspot use was calculated as the time spent by the focal individual in a patch multiplied by the mean number of individuals present, thereby accounting for asynchronous arrivals and departures.

However, the weighted approach represents a different biological measure from the original hotspot definition. While the original metric quantifies prolonged individual use of locations, which are assumed to be more valuable based on optimal foraging theory, the weighted metric captures the effects of resource depletion on hotspot identification. Furthermore, because time in association was a predictor in our models, weighting hotspot use by the number of individuals present may introduce partial dependence between the response and predictor, as individuals spending more time in association will inherently contribute to higher weighted hotspot values.

We then compared the effects of associations on the number of hotspots visited per day and time spent in hotspots using the original and weighted hotspot metric, at the population level. Results differed between the two approaches. Using the original hotspot definition, time spent in association was negatively associated with the number of hotspots visited (estimate = -0.133 ± 0.026 SE, z = -5.22, p < 0.001) and with time spent in hotspots (estimate = -0.041 ± 0.011 SE, t = -3.64), suggesting that individuals spending more time in association visited fewer hotspots and spent less time at these locations.

In contrast, the weighted hotspot metric identified a substantially larger number of hotspots (n= 12158) compared to the original method (n=6508). The analyses on the effects of time in association produced a strong positive association between time spent in association and the number of weighted hotspots visited (estimate = 0.883 ± 0.025 SE, z = 35.24, p < 0.001). In the corresponding analysis on weighted time spent in hotspot, the association remained negative but was substantially stronger than in the original analysis (estimate = -0.713 ± 0.014 SE, t = -51.99). The former result may indicate that individuals identify a larger number of hotspots when in association, potentially due to pooled spatial knowledge. However, this effect may also be an artifact of the response variable (weighted time spent in hotspot) being partially dependent on the predictor (time spent in association). The latter result reinforces our original finding of faster resource depletion in association but is subject to the same statistical limitation.
